# Genetic diversity of the Chinese goat in the littoral zone of the Yangtze River as assessed by microsatellite and mtDNA

**DOI:** 10.1002/ece3.4100

**Published:** 2018-04-24

**Authors:** Guang‐Xin E, Yong‐Ju Zhao, Li‐Peng Chen, Yue‐Hui Ma, Ming‐Xing Chu, Xiang‐Long Li, Qiong‐Hua Hong, Lan‐Hui Li, Ji‐Jun Guo, Lan Zhu, Yan‐Guo Han, Hui‐Jiang Gao, Jia‐Hua Zhang, Huai‐Zhi Jiang, Cao‐De Jiang, Gao‐Fu Wang, Hang‐Xing Ren, Mei‐Lan Jin, Yuan‐Zhi Sun, Peng Zhou, Yong‐Fu Huang

**Affiliations:** ^1^ College of Animal Science and Technology, Chongqing Key Laboratory of Forage & Herbivore Chongqing Engineering Research Centre for Herbivores Resource Protection and Utilization Southwest University Chongqing China; ^2^ State Key Laboratory of Genetic Resources and Evolution Kunming Institute of Zoology Chinese Academy of Sciences Kunming China; ^3^ Institute of Animal Science Chinese Academy of Agricultural Sciences (CAAS) Beijing China; ^4^ College of Animal Science and Technology Hebei Normal University of Science & Technology Qinghuangdao China; ^5^ Yunnan Animal Scinence and Veterinary Institute Kunming China; ^6^ College of Animal Science and Technology Agricultural University of Hebei Baoding, Hebei China; ^7^ Animal Husbandry Station of Qinghai Province Xining, Qinghai China; ^8^ Animal Science and Technology College Jilin Agriculture University Changchun, Jilin China; ^9^ Chongqing Academy of Animal Sciences Chongqing China; ^10^ Wuhan Tianyi Huiyuan Bioscience & Technology Inc Wuhan China

**Keywords:** diversity, goat, microsatellite, mitochondrial DNA, Yangtze River

## Abstract

The objective of this study was to assess the genetic diversity and population structure of goats in the Yangtze River region using microsatellite and mtDNA to better understand the current status of those goat genetic diversity and the effects of natural landscape in fashion of domestic animal genetic diversity. The genetic variability of 16 goat populations in the littoral zone of the Yangtze River was estimated using 21 autosomal microsatellites, which revealed high diversity and genetic population clustering with a dispersed geographical distribution. A phylogenetic analysis of the mitochondrial D‐loop region (482 bp) was conducted in 494 goats from the Yangtze River region. In total, 117 SNPs were reconstructed, and 173 haplotypes were identified, 94.5% of which belonged to lineages A and B. Lineages C, D, and G had lower frequencies (5.2%), and lineage F haplotypes were undetected. Several high‐frequency haplotypes were shared by different ecogeographically distributed populations, and the close phylogenetic relationships among certain low‐frequency haplotypes indicated the historical exchange of genetic material among these populations. In particular, the lineage G haplotype suggests that some west Asian goat genetic material may have been transferred to China via Muslim migration.

## 1 INTRODUCTION

The goat (*Capra hircus*) is a culturally and economically important domestic animal worldwide. The goat is also one of the major landmarks in the evolution of human modes of subsistence (Doro et al., 2014; Zeder, 2008).

To date, the global diversity of local goats has been assessed using various molecular tools, and such studies have been conducted in Spain (Ferrando et al., 2015; Manunza et al., 2016), Africa (Awotunde et al., 2015; Kibegwa, Githui, Jung'a, Badamana, & Nyamu, 2016; Mdladla, Dzomba, Huson, & Muchadeyi, 2016), the Americas (Carvalho, Paiva, Araújo, Mariante, & Blackburn, 2015), Europe (Bulut, Kurar, Ozsensoy, Altunok, & Nizamlioglu, 2016; Windig et al., 2016), and Asia (Lin et al., 2013; Nicoloso et al., 2015; Waki, Sasazaki, Kobayashi, & Mannen, 2015; Yadav, Gahlot, Gahlot, Asraf, & Yadav, 2015).

The genetic diversity of several types of Chinese goats has also been reported, including the Chinese dairy goat (Wang et al., 2015), goats indigenous to southwest China (Wei et al., 2014; Zhao et al., 2014), the black goat (Miao et al., 2015; Zhong et al., 2013), and the cashmere goat (Di et al., 2011; Liu et al., 2013).

Most studies primarily consider a relatively small group of breeds within an administrative area; however, due to geographical isolation and adaptation to different nutrient supplies and climates, the population structure always follows the geographical distribution and management history (Ling et al., 2012; Yadav et al., 2015). Human social activities play an equally important role in shaping genetic diversity and the evolution of the population genetic structure of domestic animals.

The Yangtze River, which is the longest river in Asia and the third longest river in the world, plays an important role in the archeology, civilization, and economy of China, particularly in the upstream region of the Yangtze River (Jin et al., 2012). For thousands of years, the Yangtze River has been used for transportation and boundary‐marking. Moreover, due to a multitier transport network comprising water, railways, and roads, a new economic belt was created alongside the river. This transport network has driven a new round of human mass migration and cultural exchange. In addition, the upstream region of the Yangtze River is the origin of indigenous domesticated goats and human civilization in China (China National Commission of Animal Genetic Resources, 2011).

In this study, we estimated the molecular genetic diversity and population phylogenetic structure of domestic goat populations in the upstream region of the Yangtze River using microsatellites and D‐loop DNA to better understand that Yangtze River, as natural landscape and carrier of human civilization development, what is its influence in shaping the genetic diversity of domestic animal. In addition, the aim of this research was also to identify the diversity among breeds and populations, and provide basis for genetic resources conservation strategy in native goats in this area.

## 2 MATERIAL AND METHODS

### 2.1 Animals and microsatellite marker methods

The experimental conditions in this study were approved by the Committee on the Ethics of Animal Experiments of Southwest University (No. [2007] 3) and the Animal Protection Law of China. In total, 426 individual blood samples from goats in 16 populations in the littoral zone of the Yangtze River (Table 1) were collected in EDTA tubes, and a standard phenol:chloroform protocol (Sambrook & Russell, 2001) was used to extract genomic DNA; a 0.8% agarose gel was used to assess the DNA quality, and a DTX microplate reader (Beckman Coulter, US) was used to quantify the extracted DNA.

**Table 1:** Sampling information of sixteen goat populations in the littoral zone of the Yangtze River

Name	Code	Sample size	North latitude	East longitude	Geographical location
Hechuan white goat	HW	24	29°58′29.98″	106°16′21.20″	Hechuan, Chongqing, China
Dazu black goat	DZ	38	29°39′26.25″	105°44′14.97″	Dazu, Chongqing, China
Banjiao goat	BJ	24	31°56′59.82″	108°39′35.84″	Chenkou, Chongqing, China
Youzhou black‐skin goat	UW	25	28°50′39.76″	108°45′48.46″	Youyang, Chongqing, China
Meigu goat	MG	34	28°26′25.99″	103°05′35.76″	Meigu, Sichuan, China
Chuannan black goat	CN	31	28°46′0.95″	104°37′27.32″	Luzhou, Sichuan, China
Jianyang big ear goat	JY	30	30°23′22.17″	104°31′38.75″	Jianzhou, Sichuan, China
Chengdu Ma goat	CDM	30	30°34′21.43″	104°03′44.18″	Chengdu, Sichuan, China
Yunling goat	YL	15	25°43′29.15″	101°19′22.03″	Dayao, Yunnan, China
Zhaotong goat	ZT	26	27°20′29.48″	103°42′53.04″	Zhaotong, Yunnan, China
Yichang white goat	YW	24	30°43′46.09″	111°17′55.27″	Yichang, Hubei, China
Black‐bone goat	WG	24	30°35′42.60″	114°17′59.29″	Wuhan, Hubei, China
Enshi black goat	EB	24	30°16′57.55″	109°28′49.61″	Enshi, Hubei, China
Chaidamu Cashmere goat	CM	26	35°29′40.31″	96°11′43.09″	Haixi, Qinghai, China
Qinghai Tibetan goat	QH	26	36°37′2.27″	101°46′33.24″	Xining, Qinghai, China
Nubia (Introduced)	NB	25	31°09′47.02″	108°23′18.55″	Kaizhou (from Australia), Chongqing, China

All goats were genotyped using twenty‐one microsatellite markers (Table 2) as recommended by FAO (FAO, 2011). The genotyping was conducted using PCR protocols and a 3130xl Genetic Analyzer (Applied Bio Systems) as previously described by E et al. (2016).

**Table 2:** Primer information for twenty‐one microsatellites as recommended by the FAO

Locus	Primer	Sequences (5′–3′)	Chromosomal location	Annealing temp. (°C)	Allele range (bp)
CSRD247	Forward	GGACTTGCCAGAACTCTGCAAT	OAR14	58	220–247
	Reverse	CACTGTGGTTTGTATTAGTCAGG			
TCRVB6	Forward	GAGTCCTCAGCAAGCAGGTC	BTA10	55	217–255
	Reverse	CCAGGAATTGGATCACACCT			
MAF209	Forward	GATCACAAAAAGTTGGATACAACCGTG	CHI17	55	100–104
	Reverse	TCATGCACTTAAGTATGTAGGATGCTG			
SRCRSP113	Forward	CCTCCACACAGGCTTCTCTGACTT	BTA10	58	134–158
	Reverse	CCTAACTTGCTTGAGTTATTGCCC			
SRCRSP5	Forward	GGACTCTACCAACTGAGCTACAAG	CHI21	55	156–178
	Reverse	TGAAATGAAGCTAAAGCAATGC			
SRCRSP8	Forward	TGCGGTCTGGTTCTGATTTCAC	Unknown	55	215–255
	Reverse	GTTTCTTCCTGCATGAGAAAGTCGATGCTTAG			
MAF065	Forward	AAAGGCCAGAGTATGCAATTAGGAG	OAR15	58	116–158
	Reverse	CCACTCCTCCTGAGAATATAACATG			
INRA063	Forward	GACCACAAAGGGATTTGCACAAGC	CHI18	58	164–186
	Reverse	AAACCACAGAAATGCTTGGAAG			
MAF70	Forward	CACGGAGTCACAAAGAGTCAGACC	BTA4	65	134–168
	Reverse	GCAGGACTCTACGGGGCCTTTGC			
OarFCB48	Forward	GAGTTAGTACAAGGATGACAAGAGGCAC	OAR17	58	149–173
	Reverse	GACTCTAGAGGATCGCAAAGAACCAG			
INRA023	Forward	GAGTAGAGCTACAAGATAAACTTC	BTA3	58	196–215
	Reverse	TAACTACAGGGTGTTAGATGAACT			
SRCRSP9	Forward	AGAGGATCTGGAAATGGAATC	CHI12	58	99–135
	Reverse	GCACTCTTTTCAGCCCTAATG			
OarAE54	Forward	TACTAAAGAAACATGAAGCTCCCA	OAR25	58	115–138
	Reverse	GGAAACATTTATTCTTATTCCTCAGTG			
OarFCB20	Forward	GGAAAACCCCCATATATACCTATAC	OAR2	58	93–112
	Reverse	AAATGTGTTTAAGATTCCATACATGTG			
ILSTS011	Forward	GCTTGCTACATGGAAAGTGC	BTA14	58	250–300
	Reverse	CTAAAATGCAGAGCCCTACC			
ILSTS005	Forward	GGAAGCAATTGAAATCTATAGCC	BTA10	55	172–218
	Reverse	TGTTCTGTGAGTTTGTAAGC			
SRCRSP15	Forward	CTTTACTTCTGACATGGTATTTCC	Unknown	55	172–198
	Reverse	TGCCACTCAATTTAGCAAGC			
ILSTS029	Forward	TGTTTTGATGGAACACAG	BTA3	55	148–170
	Reverse	TGGATTTAGACCAGGGTTGG			
TGLA53	Forward	GCTTTCAGAAATAGTTTGCATTCA	BTA16	55	126–160
	Reverse	ATCTTCACATGATATTACAGCAGA			
INRABERN185	Forward	CAATCTTGCTCCCACTATGC	CHI18	55	261–289
	Reverse	CTCCTAAAACACTCCCACACTA			
SRCRSP7	Forward	TCTCAGCACCTTAATTGCTCT	CHI6	55	117–131
	Reverse	GGTCAACACTCCAATGGTGAG			

The mean number of alleles (*N*_A_), observed (*H*_o_) heterozygosity, expected heterozygosity (*H*_E_), polymorphism information content (PIC), and inbreeding coefficient (*F*_IS_) were estimated using FSTAT 2.9.3.2 (Goudet, 1995) and Microsatellite Toolkit software (Park, 2001). To identify possible deviations from Hardy–Weinberg equilibrium (HWE), we used Fisher's exact test with Bonferroni correction using GENEPOP 3.4 software (Raymond & Rousset, 1995). Pairwise differences in populations (*F*_ST_) (Slatkin, 1995) were identified using R‐lequin software version 3.5.1.3 (Excoffier & Lischer, 2010). Phylogenetic neighbor‐joining trees were derived from the Reynolds genetic distance using the PHYLIP software package (Felsenstein, 2005). The Reynolds genetic distance among populations was visualized using a Neighbor‐Net generated using SPLITSTREE4 software (Huson & Bryant, 2006). The Bayesian clustering algorithm was implemented in STRUCTURE 2.3.3 (Falush, Stephens, & Pritchard, 2003) to determine the population structure and explore the assignment of individuals and populations to specific gene clusters using a burn‐in of 50 000, followed by 100,000 Markov Chain Monte Carlo (MCMC) iterations from *K = *1 to *K = *16 in 100 runs, and a merger of these runs within each of the *K* values obtained from CLUMPP software (Jakobsson & Rosenberg, 2007) was visualized using DISTRUCT 1.1 software (Rosenberg, 2004). A graphical display of the simulated results and the optimal *K* value were generated using STRUCTURE_Harvester (Earl & vonHoldt, 2012).

### 2.2 Animals and experimental methods using mtDNA D‐loop variants

The mtDNA D‐loop variants of 326 related goats are shown in Table 6. The high‐variability region in the mtDNA control region was amplified using the primers CAP‐F (5′‐CGTGTATGCAATGACATAC‐3′) and CAP‐R (5′‐CTGATTAGTCATTAGTCCATC‐3′) (Zhao et al., 2014). The PCR amplification was conducted using a Bio‐RAD T100^™^ Thermal Cycler (Bio‐Rad Laboratories Pty. Ltd, New South Wales, Australia) with a total reaction volume of 50 μl, containing 150 ng of DNA, 25 μl of 2× PCR‐Mix (BioMed, Beijing, China), 20 pmol/L each of the forward and reverse primer, and ddH_2_O to bring the volume up to 50 μl. The PCR protocol was as follows: an initial denaturation step at 95°C for 3 min, followed by 28 cycles of 95°C for 30 s, 58°C for 30 s, and 72°C for 1 min. The final cycle was followed by a 72°C extension for 10 min. Tanyibiotech (Wuhan, China) performed the sequencing using CAP‐F.

The sequence alignments of the D‐loop region in all goats were constructed using Clustal X software (1.83) (Cummings, Neel, & Shaw, 2008) with GenBank No. AF533441 as the reference sequence. DnaSP5.10 (Librado & Rozas, 2009) was conducted to screen for haplotypes and polymorphisms. Bayesian inference (BI) and maximum likelihood (ML) frameworks were used to examine the phylogenetic relationships. The best fitting model of DNA substitution for BI (the Hasegawa–Kishino–Yano model (G + I)) was obtained using jModelTest V. 0.1.1. software (Posada, 2008). The neighbor‐joining phylogenetic network was rebuilt using MEGA (6.0) software (Tamura, Stecher, Peterson, Filipski, & Kumar, 2013), and the bootstrap values to support the nodes of the tree were based on 1,000 iterations of the heuristic search. Twelve reference sequences (lineage A (AY155721, EF618134, and EF617965), lineage B (EF617965 and DQ121578), lineage C (AY155708 and DQ188892), lineage D (AY155952 and DQ188893), lineage F (DQ241349 and DQ241351), and lineage G (EF618084)) from a previous study (Naderi et al., 2007) were used in this study. All sequences generated in this study were deposited in the GenBank database (KX660779‐1003, KU891395‐494).

## 3 RESULTS

### 3.1 Diversity estimation and population structure of Chinese goat in the littoral zone of the Yangtze River using microsatellite analysis

Across all 16 goat populations in the littoral zone of the Yangtze River (see Table 1), 435 alleles were found by analyzing 21 microsatellite markers (Table 2). On average, 23 alleles per marker were observed, with a minimum of six alleles in MAF209 and a maximum of 46 alleles in INRA023. The average *H*_O_ and *H*_E_ within the markers in all populations were 0.521 (0.237 (ILSTS005) to 0.698 (MAF065) and 0.640 (0.399 (ILSTS029) to 0.768 (SRCRSP5)), respectively. The average PIC across the markers was 0.590, which ranged from 0.364 (ILSTS029) to 0.721 (SRCRSP5) (Table 3, Appendice S1).

**Table 3:** Genetic diversity of 21 microsatellite locus across sixteen goat populations in the littoral zone of the Yangtze River

Locus	*H*_O_	*H*_E_	PIC	*N*_A_	dHWE‐Q	Pa
INRA023	0.611	0.755	0.707	46	10	19
ILSTS005	0.237	0.554	0.495	16	14	5
INRABERN185	0.445	0.488	0.450	23	6	9
MAF065	0.698	0.753	0.708	21	4	7
INRA063	0.646	0.709	0.655	20	6	8
ILSTS011	0.531	0.622	0.576	23	8	3
OarFCB20	0.604	0.668	0.614	21	6	6
SRCRSP7	0.246	0.502	0.445	16	14	5
ILSTS029	0.279	0.399	0.364	24	6	8
SPS113	0.644	0.720	0.661	21	10	7
CSRD247	0.638	0.758	0.715	26	10	6
SRCRSP5	0.616	0.768	0.721	25	7	4
MAF209	0.309	0.447	0.366	6	5	1
SRCRSP8	0.496	0.734	0.688	27	12	5
SRCRSP9	0.655	0.694	0.637	23	11	6
SRCRSP15	0.382	0.456	0.408	20	5	9
TCRVB6	0.601	0.716	0.668	29	11	3
MAF70	0.446	0.619	0.571	21	9	8
OarFCB48	0.614	0.690	0.635	25	2	6
OarAE54	0.634	0.743	0.695	21	6	4
TGLA53	0.616	0.654	0.604	27	4	9
Mean	0.521	0.640	0.590	23	7.90	6.57

dHWE‐Q is number of populations deviated from Hardy–Weinberg equilibrium in this marker. Pa is number of private alleles.

Across all markers, the *N*_A_ value ranged from 4.857 ± 1.878 in the QH population to 10.476 ± 4,729 in the MG population. The *H*_O_ value ranged from 0.420 ± 0.020 in the CN population to 0.623 ± 0.021 in the CM populations, and the *H*_E_ value ranged from 0.569 ± 0.046 in the EB population to 0.743 ± 0.024 in the MG population (Table 4).

**Table 4:** Diversity estimation of sixteen populations in the littoral zone of the Yangtze River using microsatellite markers

Population	*H*_O_ (±*SD*)	*H*_E_ (±*SD*)	*N*_A_ (±*SD*)	*F*_IS_	*p*‐Value	dHWE‐M	Pa
BJ	0.557 ± 0.022	0.635 ± 0.044	6.048 ± 1.987	0.126	.0001*	5	1
CDM	0.503 ± 0.018	0.583 ± 0.050	6.524 ± 2.804	0.139	.0001*	8	1
CM	0.623 ± 0.021	0.738 ± 0.029	8.429 ± 2.619	0.158	.0001*	9	15
CN	0.420 ± 0.020	0.654 ± 0.030	7.524 ± 2.522	0.362	.0001*	15	7
DZ	0.598 ± 0.022	0.660 ± 0.031	6.190 ± 2.786	0.096	.0003	7	4
EB	0.450 ± 0.022	0.569 ± 0.046	5.857 ± 2.581	0.213	.0001*	12	4
HW	0.578 ± 0.022	0.606 ± 0.038	6.429 ± 3.249	0.047	.0436	7	6
JY	0.493 ± 0.021	0.693 ± 0.043	8.571 ± 3.280	0.292	.0001*	17	19
MG	0.444 ± 0.020	0.743 ± 0.024	10.476 ± 4.729	0.407	.0001*	18	31
NB	0.610 ± 0.021	0.643 ± 0.041	6.429 ± 2.619	0.052	.0177	6	3
QH	0.549 ± 0.021	0.619 ± 0.035	4.857 ± 1.878	0.114	.0001*	18	4
UW	0.569 ± 0.022	0.610 ± 0.034	5.667 ± 2.033	0.068	.0068	5	2
WG	0.484 ± 0.022	0.606 ± 0.033	5.095 ± 1.670	0.204	.0001*	7	4
YW	0.464 ± 0.022	0.677 ± 0.039	7.857 ± 3.214	0.320	.0001*	12	14
YL	0.500 ± 0.019	0.600 ± 0.045	6.190 ± 2.839	0.168	.0001*	10	8
ZT	0.500 ± 0.021	0.611 ± 0.036	6.381 ± 2.765	0.185	.0001*	10	2

Pa is number of private alleles, dHWE‐M is number of marker deviated from Hardy–Weinberg equilibrium within population.

Indicative adjusted nominal level (5%) for one table is 0.00015 based on 6,720 randomizations of *p*‐value for *F*_IS_, and “*” mean the significance *p*‐value of *F*_IS_.

On average, each locus deviated from HWE in 7.90 populations. The most extreme markers, that is, ILSTS005 and SRCRSP7, deviated from HWE in 14 populations (Table 3). In addition, the number of markers deviated from HWE within populations by 5 (BJ and UW) to 18 (MG and QH), and no population that lacked the expected marker deviated from HWE (Table 4).

The *F*_IS_ within a population ranged from 0.047 in the HW population to 0.407 in the MG population. The *F*_IS_ was below 0.1 in four populations (i.e., HW, NB, UW, and DZ) and was above 0.20 in six populations (i.e., WG, EB, JY, YW, CN, and MG). Most of the populations (12/16) had a significant *p*‐value for *F*_IS_.

In total, 125 private alleles were distributed across 16 populations and 21 microsatellite markers. The frequency of certain private alleles was high in certain populations. For example, the frequency of the 208‐bp allele SRCRSP15 was 30.43% in the YW population, and the frequencies of the 193 and 194 bp alleles INRA023 were 8.82% and 13.24% in the MG populations, respectively. The 156‐bp allele SPS113 had a frequency of 17.24% in the QH population, and the 217‐bp allele CSRD247 had a frequency of 22.92% in the WG (Table 4 and Appendix S1). Among all populations, the mean number of private alleles was 6.57 per marker, and the range was from 1 (MAF209) to 19 (INRA023).

**Figure 1:**
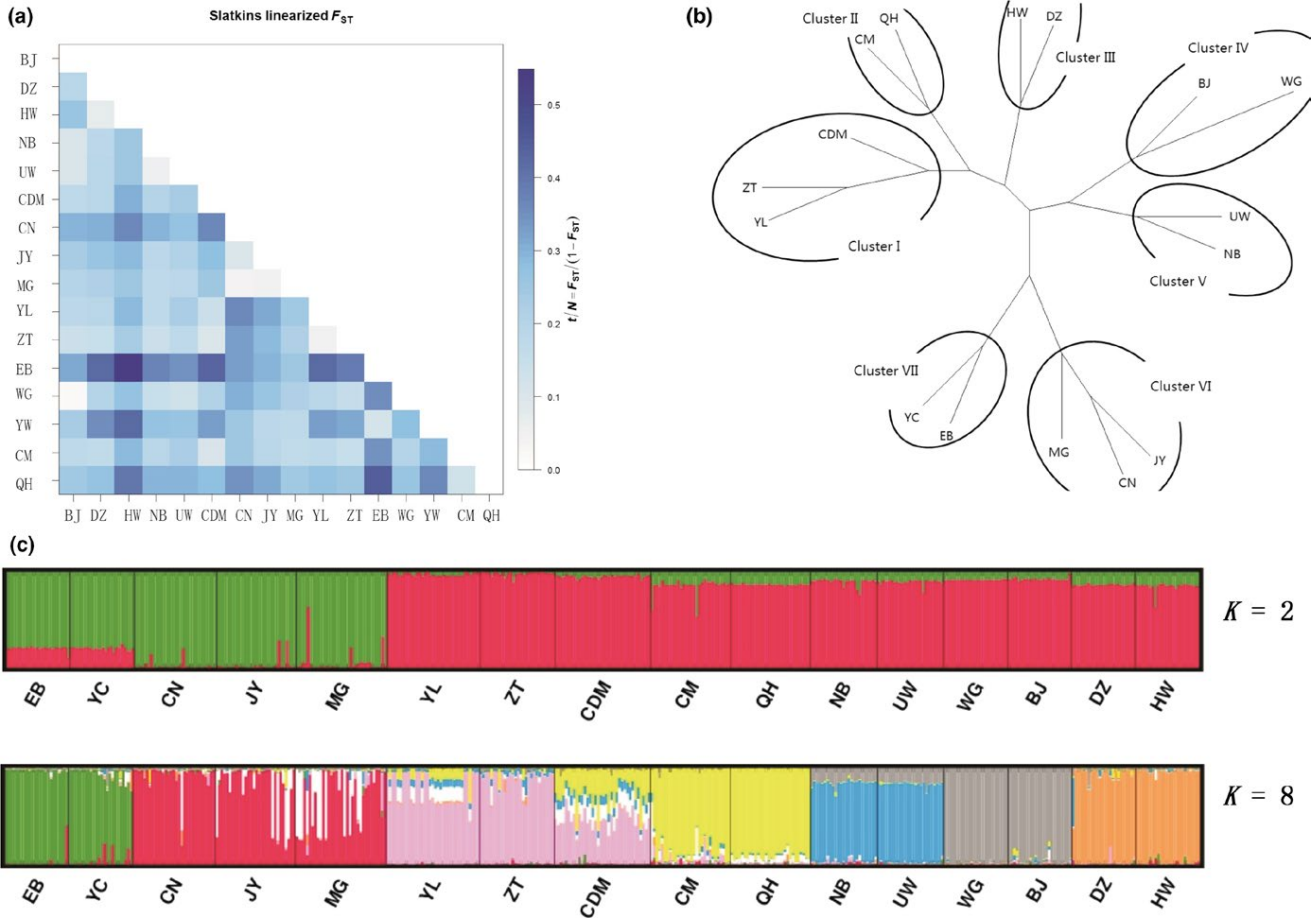
Phylogenetic population structure of sixteen goat populations in the littoral zone of the Yangtze River using microsatellite markers. (a). Matrix of the Slatkin‐linearized *F*_ST_ values as *t*/*N* = *F*_ST_/(1 − *F*_ST_) among sixteen goat populations. (b) Phylogenetic network tree of 16 goat populations derived from Reynold's genetic distance. (c) Cluster diagrams of 16 goat populations obtained from the optimal K‐ values, i.e., *K *=* *2 and *K *=* *8, and the optimal *K* value as indicated by Delta *K* = m|L″(*K*)|/s|L(*K*)|

In the *F*_ST_ analysis, the greatest divergence was observed between HW and EB (0.549), and the smallest divergence was observed between WG and BJ (0.025, Table 5). Thus, the distribution of *F*_ST_ did not reveal a significant divergence among different ecogeographically distributed populations. However, the populations in closer distribution locations, particularly those in southwest China (i.e., Chongqing, Sichuan, and Yunnan), had a significantly lower divergence compared with that in populations from geographically distant populations (Table 5, Figure 1a).

**Table 5:** Pairwise differences in population averages (Slatkins linearized *F*_ST_) using microsatellite markers

	BJ	CDM	CM	CN	DZ	EB	HW	JY	MG	NB	QH	UW	WG	YW	YL	ZT
BJ	0.000															
CDM	0.179	0.000														
CM	0.172	0.106	0.000													
CN	0.295	0.361	0.256	0.000												
DZ	0.189	0.195	0.164	0.308	0.000											
EB	0.312	0.433	0.351	0.332	0.420	0.000										
HW	0.265	0.305	0.288	0.367	0.069	0.549	0.000									
JY	0.233	0.276	0.197	0.096	0.258	0.292	0.288	0.000								
MG	0.201	0.253	0.167	0.041	0.215	0.253	0.250	0.050	0.000							
NB	0.108	0.204	0.193	0.294	0.186	0.372	0.252	0.182	0.174	0.000						
QH	0.246	0.276	0.131	0.350	0.268	0.448	0.396	0.310	0.243	0.295	0.000					
UW	0.110	0.240	0.228	0.272	0.193	0.351	0.252	0.209	0.183	0.056	0.299	0.000				
WG	0.025	0.213	0.204	0.301	0.202	0.356	0.272	0.261	0.217	0.151	0.263	0.129	0.000			
YW	0.237	0.333	0.286	0.255	0.353	0.116	0.421	0.188	0.175	0.272	0.365	0.263	0.279	0.000		
YL	0.182	0.143	0.178	0.367	0.192	0.426	0.286	0.310	0.244	0.180	0.270	0.223	0.194	0.326	0.000	
ZT	0.138	0.100	0.149	0.333	0.148	0.388	0.233	0.289	0.222	0.145	0.257	0.180	0.156	0.312	0.051	0.000

“*” Mean the significance *p*‐value (significance level = .0500) of variance analysis.

The phylogenetic neighbor‐joining network of the sixteen goat populations shows seven subclusters. These subclusters include the ZT, CDM, and YL populations, which were separated into cluster I; the CM and QH populations, which were sampled in the Qinghai (Tibetan region) and separated into Cluster II; the DZ and HW populations, which were collected in Chongqing and separated into Cluster III; the BJ (from Chongqing) and WG (from Yunnan) populations, which were separated into Cluster IV; the UW and NB populations, which were separated into Cluster V; the JY, CN and MG populations, which were sampled in Sichuan and separated into Cluster VI; and the EB and YW populations, which were sampled in Hubei and separated into Cluster VII, (Figure 1b).

The clustering of individuals into 1 ≤ *K *≤* *16 was estimated using the STRUCTURE software by setting the optimal *K* value to 2 and 8 (Appendice S2). At *K *=* *2, the populations were separated into two different groups; the first group comprised YW, EB, MG, CN, and JY, and the other group comprised the remaining populations. At *K *=* *8, the population structure was separated into seven group, which was similar to the groups separated using the neighbor‐joining tree. The population structure analysis using both STRUCTURE and the phylogenetic neighbor‐joining network revealed a population structure without a unanimous ecogeographical distribution (Figure 1c).

### 3.2 Diversity estimation and population structure of Chinese goat in the littoral zone of the Yangtze River using mtDNA D‐loop

To analyze the mtDNA D‐loops of domestic goats in the littoral zone of the Yangtze river, samples from 326 goats from 13 populations were obtained, and the sequences were 582 bp in length as published in the GenBank database (Table 6). A meta‐analysis of 168 mtDNA D‐loop sequences from nine other populations was conducted using previous reports and data obtained in this study to generate a larger sample size (Table 6). In total, 494 sample sequences (452 bp in length from 15,737 to 16,188 as AF533441) were used for further analyses.

**Table 6:** Population name, geographical location, and DNA GenBank number for meta‐analysis using the D‐loop region of mitochondrial DNA

Population name	Code	Sampling location	Sample size	Accession numbers
Banjiao^A^	BJ	Wulong, Chongqing, China	30	KX660779‐806
Chengduma^A^	CDM	Chengdu, Sichuan, China	22	KX660807‐28
Chaidamu cashmere^A^	CM	Chaidamu, Qinghai, China	30	KX660829‐58
Dazu black^A^	DZ	Dazu, Chongqing, China	16	KU891479‐94
Fengqing^A^	FQ	Fengqing, Yunnan, China	29	KX660859‐87
Fushun^A^	FS	Zigong, Sichuan, China	17	KU891424‐40
Honggu^A^	HG	Nile, Yunnan, China	26	KX660888‐913
Hechuan white^A^	HW	Hechuan, Chongqing, China	18	KU891441‐58
Qinghai Tibetan^A^	QH	Xining, Qinghai, China	30	KX660944‐73
Youzhou black skin^A^	UW	Youyang, Chongqing, China	20	KU891459‐78
Wugu^A^	WG	Wuhan, Hubei, China	30	KX660974‐1003
Matou^A^	MT	Yunnan, China	30	KX660914‐43
Nubia (Introduced)^A^	NB	Kaizhou, Chongqing, China	28	KU891395‐23
Qianbeima^B^	QB	Guizhou, China	13	DQ121591‐603
Chuandong white^B^	CDW	Chongqing, China	19	AY860880‐84, DQ089209‐22
Hunan matou^B^	MTH	Xinhuang, Hunan, China	26	DQ121577‐90. DQ089269‐80
Yichang white^B^	YC	Yichang, Hubei, China	13	DQ089246‐58
Nanjiang yellow^B^	NJ	Sichuan, China	14	AY860920‐33
Guizhou black^C^	GZB	Guizhou, China	23	DQ121521‐34, DQ089237‐45
Yunling^C^	YLB	Yunling, Yunnan, China	24	DQ089381‐404
Guishan^C^	GS	Yunnan, China	21	DQ089160‐77, HQ199110, HQ199169‐70
Yingshan^C^	YS	Sichuan, China	15	HQ199159‐68, HQ199198‐202, DQ121591
Total	22		494	

Superscript A means the data from this study, B means the sequences cited from Liu et al. (2009), and C means the sequences cited from Zhong et al. (2013).

**Figure 2:**
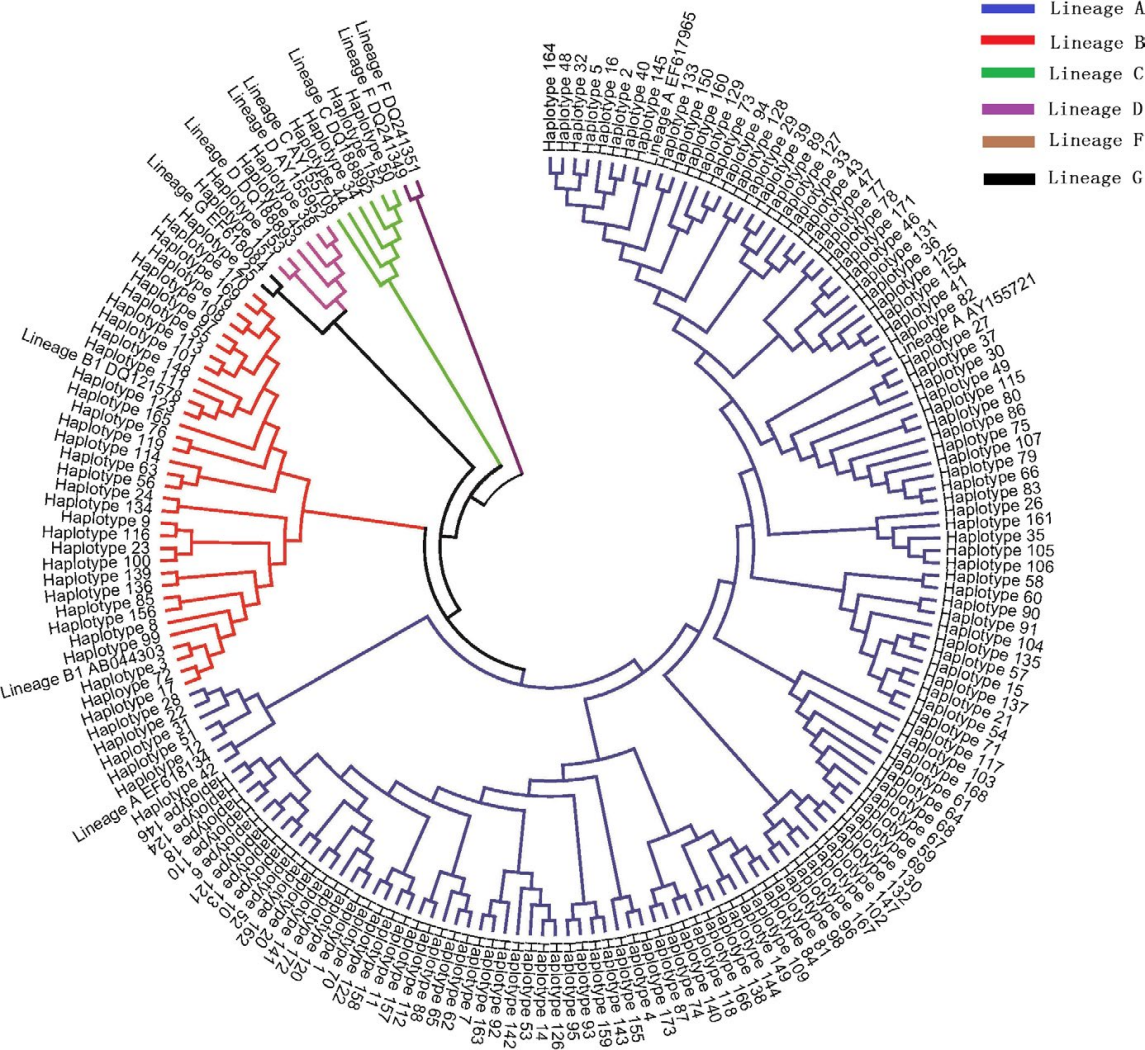
Phylogenetic relationship of mtDNA D‐loop haplotype of 173 haplotypes from 494 domestic goats in the Littoral Zone of the Yangtze River. Note. NJ tree drown by the bootstrap values was based on 1,000 iterations with Hasegawa‐Kishino‐Yano model (G + I)

We found 117 single‐nucleotide polymorphism (SNP) sites across the 452‐bp alignment sequence, and no insertions/deletions were identified (Appendice S3). One hundred and seventy‐three haplotypes were identified in 494 samples from 21 goat populations in the littoral zone of the Yangtze River (Figure 2, Appendice S4). According to the known goat lineage grouping system (Naderi et al., 2007), 132 haplotypes belonged to lineage A, 32 haplotypes belonged to lineage B, four haplotypes belonged to lineage C, four haplotypes belonged to lineage D, one haplotype belonged to lineage G, and no haplotypes were similar to those in lineage F.

The distribution of the haplotypes, such as haplotype 1 and haplotype 3, was shared by different geographical populations. However, most low‐frequency haplotypes, such as haplotypes 32–38 in the CM population and haplotypes 56–59 in the FQ population, were private. Haplotypes 150–152 had a high frequency within the QH population. The frequency of each haplotype is shown in Appendix S2.

## 4 DISCUSSION

In this study, high heterozygosity levels (*H*_E_ and *H*_O_), PIC, and *N*_A_ were observed in all microsatellite markers across 16 goat populations, indicating that these markers were adequate for estimating diversity within each population. In addition, several markers showed a deviation from HWE and allele privatization, which could have been due to the small sample size or the possible occurrence of recent population genetic events.

Using 21 markers in sixteen goat populations, a high diversity was observed within the populations according to the *H*_O_, *H*_E_, and *N*_A_ values. However, the *H*_O_ value in each population was lower than the *H*_E_ value, and 5–18 markers deviated from HWE within populations. What is more, the *F*_IS_ values of 12 populations were significantly different from zero after adjusting according to the nominal level (*p *=* *.00015), suggesting that these populations could have inbreeding. The positive association between number of markers that deviated from HWE and *F*_IS_ within population (*r *=* *0.4866, *p *<* *.01) explains the deficiency of heterozygotes in populations that deviate from HWE. Of course, some other reasons could explain the deviation, including nonrandom mating, selection, genetic drift, and small population size. In this study, the populations came from different areas and all were kept in conservation farm as large populations with systematic nonrandom mating. In addition, it is well known that inbreeding occurs within populations when some individuals in large populations separate into smaller flocks or are improperly managed by humans. Thus, nonrandom mating should be the major reason for the deviation from HWE and high *F*_IS_.

In addition, the sample size, as one of reason above, in this study could not completely represent the real diversity of each population or other types of population genetic events that occurred within the goat populations. Moreover, certain high‐frequency private alleles within populations indicated that these populations could have independently undergone long‐term domestication history (Granevitze et al., 2007). Last but not least important, the null alleles could also contribute to the deviation due to incorrect genotyping, particular like 1 bp difference between the large fragments in traditional gel conditions may not sufficiently accurate to separated, which cause some different allele ignored. However, the classification platform used in this study was sufficient to identify the genotype of 1 bp differences as well known.

According to the pairwise differences (Slatkins linearized *F*_ST_) in this study, the distribution of *F*_ST_ showed low genetic divergence (not significant) among populations in general. The EB population, which is an indigenous breed of goats that live on the Enshi Grand Canyon in Hubei, was highly divergent from most populations. Thus, we inferred that this population had a lower gene flow than the other ecogeographic populations due to the specific geographical structure of their habitat. In addition, three populations (CN, JY, and MG) in Sichuan were less genetically diverge than the other populations, which could be because Sichuan is an important economic cultural center and a hub in West China, which could easily promote the gene fowl between domestic animals (China National Commission of Animal Genetic Resources, 2011).

A neighbor‐joining network used to analyze 16 goat populations in the littoral zone of the Yangtze River revealed that most populations were clustered together based on their geography, such as the two Qinghai populations (QH and CM) and the Hubei populations (EB and YC), which grouped together into Cluster II and Cluster VII, respectively. These results were consistent with many previous reports regarding the different population relationships among goats (Bulut et al., 2016). However, Cluster I and Cluster IV consisted of different ecogeographic populations, which indicated that the gene flow and migration among populations occurred with a high frequency in the Chongqing, Sichuan, and Yunnan goat populations.

The STRUCTURE analysis showed a clear clustering of these 16 goat populations, which was similar to the neighbor‐joining network pattern described above using the Reynolds genetics distance. Based on a microsatellite marker analysis, the results elucidated strong gene flow and genetic material exchange among different regions in the littoral zone of the Yangtze River, which was likely caused by human migration, commercial trade, and extensive transport during the long‐term domestication of goats.

To further discover possible evidence of gene flow and genetic material exchange among the goat populations in the upstream region of the littoral zone of the Yangtze River, the D‐loop sequences from 494 goats from 22 related populations were investigated. In total, 173 haplotypes were identified and defined by 117 SNPs, and all five known lineages, except for lineage F, were detected. Among the haplotypes, lineage A was the most frequent, followed by lineage B, at 76% and 18.5%, respectively. These results are consistent with previous studies that show that lineages A and B are dominant and prevalent in Chinese goat populations (Lin et al., 2013; Zhao et al., 2014). Four haplotypes (H_50, H_52, H_34, and H_44) from four goats were lineage C, and four haplotypes (H_38, H_45, H_153, and H_151) from nine animals were lineage D. In a previous study, lineage C and lineage D were observed to have a low frequency in Tibetan goats (Liu, Lei, Liu, & Yang, 2007); these data are consistent with our data, which showed that nearly all goats from the QH and CM populations and one goat from the DZ population belonged to lineages C and D. Previous studies have indicated that the lineage C haplotype is not found in the DZ population (Zhao et al., 2014; Zhong et al., 2013), but the lineage C haplotype has been found in the Yangtze River delta white goat (YDW, Jiangsu, China) in the littoral zone of the Yangtze River (Liu, Cao, Chen, Yao, & Liu, 2009). In addition, the lineage D haplotype, which was only observed in the QH and CM populations in this study, has been detected in goats from YDW, Inner Mongolia, and Taihang (Liu et al., 2009; Zhong et al., 2013). Furthermore, breeding records show that the CM population was bred with both the Tibetan goat and Cashmere goats, including those from the Inner Mongolia population (China National Commission of Animal Genetic Resources, 2011).

Our data show that some genetic material exchange occurs within goat populations in the Yangtze River region. An interesting haplotype, H_25 (GenBank No. KX660829), in the CM population was clustered into lineage G in this study. Lineage G has previously only been observed in goats in West Asia (Iran), the Eurasian continental shelf (Turkey), and the Asian‐African continental shelf (Egypt). This finding supports forward an inference and hypothesis that the local Qinghai goat population has experienced gene flow from abroad. This gene flow could be explained by the large‐scale migration of Muslim people into mainland China from West Asia, which resulted in the exchange of genetic material and caused the presence of lineage G in the Qinghai goat population (China National Commission of Animal Genetic Resources, 2011).

Related archeological evidence has shown that businessmen and envoys have visited central and western Asia since the Qin–Han dynasties (BC221–AD8), particularly during the Han Dynasty, and Zhang Qian built the Silk Road in the western region, which made an extraordinary contribution to the development of the economy and cultural exchange in Asia, Europe, and Africa (Muhammad, 2016). In addition, Qinghai is located in the center of China's western region and is at the intersection of three major arteries, including the Silk Road, Tang‐Fan Road, and Ancient Tea Road. Due to its vast natural pasture and cultivated land, Qinghai is not only the birthplace of many ethnic groups but is also a breeding and blood convergence ground for the development of many religions, such as Islam, which is one of the earliest religions spread in our country (Li, 2016; Yang, 2016). In particular, during the Song and Yuan Dynasties (AD 960–1368), a large number of Muslim businessmen moved to Qinghai, and until the Ming Dynasty, Islam was one of the core religions in Qinghai (Lv & Wang, 2006). Therefore, the infiltration of Muslim culture and the migration of Muslim groups not only resulted in Qinghai indigenous cultural exchange but also, unsurprisingly, in changes in agriculture and animal husbandry.

In summary, we estimated the diversity of large‐scale Chinese goat populations in the littoral zone of the Yangtze River using microsatellite and partial mtDNA D‐loop variants. These showed a high diversity level within related goat populations. According to their population structure (based on the mtDNA and microsatellite markers), there was a dispersed geographical clustering, indicating that a high frequency of genetic material exchange and gene flow among these populations of goats occurred. In particular, we discovered lineage C mtDNA in the southwestern goat populations and lineage G mtDNA in the Qinghai goat population, which was inconsistent with previous studies. Based on our results, we infer that high anthropic movement and migration related to the acclimation process of domestic animals occurred. Therefore, the results of this study are helpful for the understanding of the domestication process of domestic animals in South China and can be used to support new, planned strategies for their conservation.

## CONFLICT OF INTEREST

None declared.

## AUTHOR'S CONTRIBUTION

Yong‐Fu Huang, Guang‐Xin E, and Li‐Peng Chen participated in the experimental design and wrote the manuscript; Yong‐Ju Zhao, Ming‐Xing Chu, Yue‐Hui Ma, Jia‐Hua Zhang, Hui‐Jiang Gao, Yuan‐Zhi Sun, and Mei‐Lan Jin performed the laboratory experiment and carried out the bioinformatic data analysis; Gao‐Fu Wang, Hang‐Xing Ren, Peng Zhou, Ji‐Jun Guo, Huai‐Zhi Jiang, Lan Zhu, and Yan‐Guo Han conducted sample collection; Cao‐De Jiang, Qiong‐Hua Hong, Xiang‐Long Li, and Lan‐Hui Li have read and approved the final manuscript.
