# Supplementary material for: Genetic diversity of the Chinese goat in the littoral zone of the Yangtze River as assessed by microsatellite and mtDNA
Source: Ecol Evol. 2018 Apr 24;8(10):5111–23. doi: 10.1002/ece3.4100 (PMC5980450; doi:10.1002/ece3.4100)
Supplement: Supplementary file 1 [file ECE3-8-5111-s001.doc]

Appendix S1 The Allele frequency (%) of each microsatellite within sixteen population

| INRA023 | BJ | DZ | HW | NB | UW | CDM | CN | JY | MG | YL | ZT | EB | WG | YW | CM | QH |
| --- | --- | --- | --- | --- | --- | --- | --- | --- | --- | --- | --- | --- | --- | --- | --- | --- |
| 185bp |  |  |  |  |  |  |  |  |  | 3.23 |  |  |  |  |  |  |
| 187 bp |  |  |  |  |  | 17.14 |  |  |  | 40.32 | 8.00 |  |  |  |  |  |
| 189 bp |  |  |  |  |  |  |  |  |  | 1.61 |  |  |  |  |  |  |
| 191 bp |  |  |  |  |  |  |  |  |  | 8.06 |  |  |  |  |  |  |
| 193 bp |  |  |  |  |  |  |  |  | 8.82 |  |  |  |  |  |  |  |
| 194 bp |  |  |  |  |  |  |  |  | 13.24 |  |  |  |  |  |  |  |
| 195 bp |  |  |  |  |  | 28.57 |  | 7.14 | 4.41 |  |  |  |  |  | 21.88 | 23.53 |
| 196 bp | 33.33 |  |  | 24.00 | 62.00 |  | 21.43 | 5.36 | 30.88 | 25.81 | 40.00 | 52.27 | 54.17 | 6.52 | 6.25 |  |
| 197 bp |  |  |  |  |  | 8.57 |  | 5.36 | 5.88 |  |  |  |  |  | 28.13 |  |
| 198 bp | 16.67 |  |  | 16.00 | 2.00 |  | 7.14 | 12.50 | 4.41 | 9.68 | 22.00 |  | 2.08 |  |  |  |
| 199 bp |  |  |  | 24.00 | 26.00 | 30.00 |  | 3.57 |  | 3.23 | 10.00 |  |  |  | 18.75 | 35.29 |
| 200 bp | 16.67 |  |  |  |  |  | 53.57 | 37.50 | 7.35 | 1.61 |  | 4.55 | 33.33 | 2.17 |  |  |
| 201 bp |  |  |  | 4.00 | 2.00 | 2.86 | 3.57 | 7.14 | 4.41 |  |  |  |  |  | 3.13 |  |
| 202 bp | 10.42 |  |  |  |  |  | 3.57 | 1.79 | 2.94 |  |  |  | 2.08 |  |  |  |
| 203 bp |  |  |  | 8.00 |  | 1.43 |  |  |  |  |  |  |  |  | 6.25 | 35.29 |
| 204 bp | 2.08 |  |  |  |  |  |  |  |  |  |  |  |  | 4.35 |  |  |
| 205 bp | 4.17 |  |  | 8.00 |  | 2.86 |  | 5.36 |  |  | 6.00 |  |  | 4.35 | 12.50 |  |
| 206 bp |  |  |  |  |  |  |  | 1.79 |  |  |  |  |  |  |  |  |
| 207 bp |  |  |  | 2.00 |  |  |  |  |  | 1.61 |  |  |  |  |  | 5.88 |
| 208 bp |  |  |  |  |  |  |  |  | 1.47 |  |  |  |  |  |  |  |
| 209 bp | 12.50 |  |  | 4.00 | 6.00 | 5.71 | 7.14 |  | 4.41 |  |  | 2.27 | 8.33 | 2.17 | 3.13 |  |
| 210 bp |  |  |  |  |  |  |  |  | 4.41 |  |  |  |  |  |  |  |
| 211 bp | 2.08 |  |  | 10.00 |  | 2.86 | 1.79 | 8.93 | 2.94 | 4.84 | 12.00 |  |  |  |  |  |
| 212 bp |  |  |  |  |  |  |  |  | 1.47 |  |  |  |  |  |  |  |
| 213 bp | 2.08 |  |  |  | 2.00 |  |  |  | 1.47 |  | 2.00 |  |  |  |  |  |
| 214 bp |  |  |  |  |  |  |  |  | 1.47 |  |  |  |  |  |  |  |
| 215 bp |  |  |  |  |  |  | 1.79 |  |  |  |  |  |  |  |  |  |
| 217 bp |  |  |  |  |  |  |  | 3.57 |  |  |  |  |  |  |  |  |
| 219 bp |  |  |  |  |  |  |  |  |  |  |  | 2.27 |  | 10.87 |  |  |
| 220 bp |  | 2.08 | 4.17 |  |  |  |  |  |  |  |  |  |  |  |  |  |
| 221 bp |  | 6.25 |  |  |  |  |  |  |  |  |  |  |  |  |  |  |
| 232 bp |  |  | 2.08 |  |  |  |  |  |  |  |  | 4.55 |  | 13.04 |  |  |
| 233 bp |  |  |  |  |  |  |  |  |  |  |  | 2.27 |  | 8.70 |  |  |
| 234 bp |  | 12.50 | 4.17 |  |  |  |  |  |  |  |  | 18.18 |  | 28.26 |  |  |
| 235 bp |  | 2.08 |  |  |  |  |  |  |  |  |  |  |  |  |  |  |
| 236 bp |  | 29.17 | 62.50 |  |  |  |  |  |  |  |  | 4.55 |  |  |  |  |
| 237 bp |  | 4.17 |  |  |  |  |  |  |  |  |  |  |  | 2.17 |  |  |
| 238 bp |  |  |  |  |  |  |  |  |  |  |  | 6.82 |  |  |  |  |
| 239 bp |  |  |  |  |  |  |  |  |  |  |  |  |  | 2.17 |  |  |
| 240 bp |  | 2.08 | 2.08 |  |  |  |  |  |  |  |  |  |  | 8.70 |  |  |
| 241 bp |  |  |  |  |  |  |  |  |  |  |  |  |  | 2.17 |  |  |
| 242 bp |  | 12.50 | 2.08 |  |  |  |  |  |  |  |  | 2.27 |  | 4.35 |  |  |
| 243 bp |  | 6.25 |  |  |  |  |  |  |  |  |  |  |  |  |  |  |
| 244 bp |  | 20.83 | 20.83 |  |  |  |  |  |  |  |  |  |  |  |  |  |
| 245 bp |  | 2.08 |  |  |  |  |  |  |  |  |  |  |  |  |  |  |
| 246 bp |  |  | 2.08 |  |  |  |  |  |  |  |  |  |  |  |  |  |
| ILSTS005 | BJ | DZ | HW | NB | UW | CDM | CN | JY | MG | YL | ZT | EB | WG | YW | CM | QH |
| 171 bp |  |  |  |  |  |  | 1.61 |  |  |  |  |  |  |  |  |  |
| 175 bp |  |  |  |  |  |  |  |  | 1.47 |  |  |  |  |  |  |  |
| 176 bp |  |  |  |  |  |  |  | 1.67 |  |  |  |  |  |  | 1.92 |  |
| 177 bp | 8.33 | 20.83 | 4.76 | 8.00 | 18.00 | 12.86 | 20.97 | 8.33 | 23.53 | 38.57 | 28.57 | 12.50 | 22.92 | 12.50 | 5.77 | 83.33 |
| 178 bp |  |  | 4.76 |  |  |  | 8.06 | 5.00 | 1.47 |  |  | 4.17 |  |  |  |  |
| 179 bp |  |  |  |  |  |  |  |  |  |  |  |  |  |  | 1.92 |  |
| 180 bp |  |  |  |  |  |  |  | 1.67 | 1.47 |  |  |  |  |  |  |  |
| 181 bp |  | 66.67 | 47.62 |  |  | 82.86 | 14.52 | 15.00 | 4.41 | 25.71 | 35.71 |  |  |  | 69.23 | 16.67 |
| 182 bp | 64.58 |  | 4.76 | 74.00 | 64.00 |  | 46.77 | 55.00 | 42.65 |  |  | 72.92 | 58.33 | 43.75 |  |  |
| 183 bp |  | 12.50 | 33.33 |  |  | 4.29 | 4.84 | 1.67 | 4.41 | 35.71 | 35.71 |  |  |  | 13.46 |  |
| 184 bp | 27.08 |  | 4.76 | 18.00 | 14.00 |  | 3.23 | 6.67 | 16.18 |  |  | 10.42 | 18.75 | 43.75 |  |  |
| 185 bp |  |  |  |  | 2.00 |  |  | 1.67 |  |  |  |  |  |  |  |  |
| 186 bp |  |  |  |  |  |  |  | 3.33 | 1.47 |  |  |  |  |  |  |  |
| 187 bp |  |  |  |  | 2.00 |  |  |  |  |  |  |  |  |  |  |  |
| 188 bp |  |  |  |  |  |  |  |  | 1.47 |  |  |  |  |  | 7.69 |  |
| 189 bp |  |  |  |  |  |  |  |  | 1.47 |  |  |  |  |  |  |  |
| INRABERN185 | BJ | DZ | HW | NB | UW | CDM | CN | JY | MG | YL | ZT | EB | WG | YW | CM | QH |
| 258 bp |  |  |  |  |  |  |  |  | 2.94 |  |  |  |  |  |  |  |
| 259 bp |  |  |  |  |  |  |  |  | 2.94 |  |  |  |  |  |  |  |
| 260 bp |  |  |  |  |  |  |  |  | 7.35 |  |  |  |  |  |  |  |
| 261 bp |  |  |  |  |  |  |  |  | 1.47 |  |  |  |  |  |  |  |
| 262 bp |  |  |  |  |  |  |  | 1.72 | 5.88 |  |  |  |  |  |  |  |
| 263 bp |  |  | 2.78 |  |  |  | 79.31 | 65.52 | 51.47 |  |  | 35.42 |  | 75.00 | 2.00 | 1.79 |
| 264 bp | 2.08 |  |  | 4.00 |  | 1.39 | 3.45 | 3.45 | 4.41 |  |  |  |  |  |  | 3.57 |
| 265 bp | 77.08 | 52.17 | 44.44 | 86.00 | 76.00 | 86.11 |  | 1.72 |  | 81.82 | 89.29 |  | 66.67 |  | 58.00 | 50.00 |
| 266 bp |  |  |  |  |  |  | 1.72 |  | 1.47 |  |  |  |  |  |  |  |
| 267 bp |  |  | 5.56 |  |  | 1.39 |  |  | 1.47 | 3.03 |  |  |  |  |  |  |
| 268 bp |  |  |  |  |  |  |  | 3.45 | 1.47 |  |  |  |  |  | 2.00 |  |
| 269 bp |  |  | 2.78 |  |  |  |  | 1.72 | 1.47 |  |  |  |  |  |  |  |
| 270 bp |  |  |  |  |  |  |  | 3.45 |  |  |  |  |  |  |  |  |
| 272 bp |  |  |  |  |  |  |  | 1.72 |  |  |  |  |  |  |  |  |
| 274 bp |  |  |  |  |  |  |  |  |  |  |  |  |  |  | 2.00 | 3.57 |
| 275 bp |  |  | 2.78 |  |  |  |  | 8.62 |  |  |  | 6.25 |  | 4.17 |  |  |
| 276 bp | 6.25 | 10.87 | 2.78 |  | 6.00 | 2.78 |  |  |  |  |  |  | 6.25 |  | 12.00 |  |
| 277 bp |  |  |  |  |  | 8.33 | 5.17 | 6.90 | 10.29 | 4.55 | 1.79 | 29.17 |  | 2.08 | 16.00 | 5.36 |
| 278 bp | 4.17 | 15.22 | 22.22 | 2.00 | 8.00 |  | 3.45 |  | 1.47 |  |  |  | 6.25 | 2.08 |  | 1.79 |
| 279 bp |  | 21.74 | 16.67 | 8.00 | 10.00 |  | 6.90 |  | 2.94 | 10.61 | 8.93 | 29.17 |  | 14.58 | 8.00 | 33.93 |
| 280 bp | 10.42 |  |  |  |  |  |  |  | 2.94 |  |  |  | 20.83 |  |  |  |
| 281 bp |  |  |  |  |  |  |  |  |  |  |  |  |  | 2.08 |  |  |
| 290 bp |  |  |  |  |  |  |  | 1.72 |  |  |  |  |  |  |  |  |
| MAF065 | BJ | DZ | HW | NB | UW | CDM | CN | JY | MG | YL | ZT | EB | WG | YW | CM | QH |
| 113 bp |  |  |  |  |  |  |  |  |  |  |  |  |  |  |  | 4.00 |
| 114 bp |  |  |  |  |  |  |  | 4.35 |  |  |  |  |  |  |  |  |
| 115 bp |  |  | 2.08 |  | 6.25 |  | 1.61 | 4.35 | 2.27 |  |  |  | 2.08 | 2.08 | 1.79 | 12.00 |
| 116 bp |  |  |  |  |  |  | 3.23 |  |  |  |  |  |  |  |  |  |
| 117 bp | 22.92 | 36.96 | 45.83 | 14.00 | 33.33 | 18.57 | 29.03 | 15.22 | 18.18 | 22.58 | 44.44 | 6.25 | 37.50 | 18.75 | 17.86 |  |
| 118 bp |  |  |  |  |  |  | 3.23 |  |  |  |  |  |  |  |  |  |
| 119 bp | 18.75 | 19.57 | 18.75 | 24.00 | 16.67 | 37.14 | 16.13 | 21.74 | 11.36 | 40.32 | 25.93 | 4.17 | 6.25 | 20.83 | 48.21 | 32.00 |
| 120 bp |  |  |  |  |  |  |  |  | 11.36 |  |  |  |  |  |  |  |
| 121 bp | 22.92 | 21.74 | 16.67 | 20.00 | 18.75 | 31.43 | 14.52 | 6.52 | 15.91 | 20.97 | 12.96 | 89.58 | 27.08 | 31.25 | 5.36 | 4.00 |
| 122 bp |  |  |  |  |  |  | 3.23 | 2.17 | 2.27 |  |  |  |  |  |  |  |
| 123 bp |  |  |  | 4.00 | 6.25 |  | 6.45 |  | 18.18 |  |  |  |  |  |  |  |
| 124 bp | 2.08 | 4.35 | 8.33 |  |  |  |  |  | 4.55 |  |  |  | 14.58 |  |  |  |
| 125 bp |  |  |  |  |  | 2.86 | 11.29 | 13.04 | 4.55 | 11.29 |  |  |  | 14.58 | 5.36 | 26.00 |
| 126 bp | 20.83 | 13.04 | 8.33 | 28.00 | 12.50 | 1.43 | 1.61 | 2.17 |  | 4.84 | 12.96 |  | 6.25 |  | 1.79 |  |
| 128 bp |  |  |  |  |  | 2.86 |  |  |  |  |  |  |  |  | 3.57 | 22.00 |
| 129 bp |  |  |  |  |  |  |  | 2.17 |  |  |  |  |  |  |  |  |
| 130 bp | 6.25 |  |  |  | 6.25 |  |  |  |  |  | 1.85 |  | 4.17 | 2.08 |  |  |
| 132 bp |  | 2.17 |  | 8.00 |  |  | 4.84 | 23.91 | 9.09 |  |  |  |  | 4.17 | 7.14 |  |
| 134 bp |  |  |  |  |  | 1.43 | 3.23 |  | 2.27 |  |  |  |  |  | 8.93 |  |
| 135 bp | 4.17 |  |  |  |  |  |  |  |  |  |  |  |  |  |  |  |
| 136 bp | 2.08 | 2.17 |  | 2.00 |  | 4.29 | 1.61 | 4.35 |  |  | 1.85 |  | 2.08 | 6.25 |  |  |
| INRA063 | BJ | DZ | HW | NB | UW | CDM | CN | JY | MG | YL | ZT | EB | WG | YW | CM | QH |
| 163 bp |  |  |  |  |  |  |  | 1.67 |  |  |  |  |  |  |  |  |
| 165 bp |  |  |  |  |  |  |  |  |  |  |  |  |  |  |  | 1.67 |
| 167 bp |  |  |  |  |  |  |  | 1.67 |  |  |  |  |  |  | 1.67 |  |
| 168 bp |  |  |  |  |  |  |  |  | 1.47 |  |  |  |  |  |  |  |
| 169 bp |  |  |  |  |  |  |  | 1.67 |  |  |  |  |  |  |  |  |
| 170 bp |  |  |  |  |  |  |  | 3.33 |  |  |  |  |  |  |  |  |
| 171 bp |  |  |  |  |  |  |  | 3.33 | 1.47 |  |  |  |  |  |  |  |
| 172 bp |  |  |  | 12.00 | 4.00 | 10.00 | 9.68 | 13.33 | 11.76 | 6.06 | 28.85 | 12.50 |  | 8.33 | 5.00 | 5.00 |
| 173 bp | 10.42 | 10.42 | 29.17 |  |  |  | 3.23 |  |  |  |  | 2.08 | 2.08 |  |  |  |
| 174 bp | 50.00 | 2.08 |  | 18.00 | 58.00 | 37.14 | 51.61 | 16.67 | 26.47 | 45.45 | 34.62 | 60.42 | 62.50 | 58.33 | 8.33 |  |
| 175 bp |  | 37.50 | 37.50 |  |  |  | 4.84 |  |  |  |  |  |  |  |  |  |
| 176 bp | 16.67 | 4.17 |  | 18.00 | 18.00 | 21.43 | 14.52 | 16.67 | 17.65 | 6.06 | 7.69 | 16.67 | 18.75 | 14.58 | 20.00 | 56.67 |
| 177 bp |  | 20.83 | 22.92 |  |  |  | 3.23 | 6.67 | 5.88 |  |  |  |  |  |  |  |
| 178 bp | 10.42 | 16.67 | 10.42 | 20.00 | 14.00 | 22.86 | 9.68 | 18.33 | 8.82 | 27.27 | 1.92 | 8.33 | 10.42 | 18.75 | 30.00 | 36.67 |
| 179 bp |  |  |  |  |  |  | 1.61 |  | 7.35 |  |  |  |  |  |  |  |
| 180 bp | 12.50 | 8.33 |  | 32.00 | 6.00 | 8.57 | 1.61 | 13.33 | 11.76 | 15.15 | 26.92 |  | 6.25 |  | 35.00 |  |
| 181 bp |  |  |  |  |  |  |  |  | 2.94 |  |  |  |  |  |  |  |
| 182 bp |  |  |  |  |  |  |  | 3.33 | 1.47 |  |  |  |  |  |  |  |
| 183 bp |  |  |  |  |  |  |  |  | 1.47 |  |  |  |  |  |  |  |
| 184 bp |  |  |  |  |  |  |  |  | 1.47 |  |  |  |  |  |  |  |
| ILSTS011 | BJ | DZ | HW | NB | UW | CDM | CN | JY | MG | YL | ZT | EB | WG | YW | CM | QH |
| 261 bp |  |  |  |  |  |  |  |  | 1.47 |  |  |  |  |  |  |  |
| 262 bp |  |  |  |  |  |  |  |  |  |  |  |  |  |  | 13.33 | 33.33 |
| 264 bp |  |  |  |  |  |  |  | 3.33 | 1.47 |  |  |  |  |  |  |  |
| 265 bp |  |  |  |  |  |  |  |  |  |  |  | 2.08 |  | 2.08 |  |  |
| 266 bp |  |  |  |  |  |  |  | 1.67 |  |  |  | 4.17 |  | 14.58 |  |  |
| 267 bp |  |  |  |  | 2.00 |  |  | 3.33 | 8.82 |  |  | 2.08 |  |  |  |  |
| 268 bp | 6.25 | 2.08 |  | 10.00 | 10.00 | 37.14 |  | 1.67 |  |  |  | 2.08 | 8.33 | 14.58 |  |  |
| 269 bp |  | 2.08 | 2.08 |  |  |  | 6.45 | 6.67 | 10.29 |  |  |  |  |  |  |  |
| 270 bp | 27.08 |  |  | 8.00 | 20.00 | 1.43 |  |  | 1.47 | 12.86 | 27.78 | 2.08 | 18.75 | 6.25 | 6.67 | 28.33 |
| 271 bp |  | 6.25 |  |  |  |  |  |  | 2.94 |  |  |  |  |  |  |  |
| 272 bp | 2.08 | 2.08 |  |  |  | 1.43 |  | 1.67 |  | 15.71 | 1.85 |  | 6.25 | 2.08 | 1.67 | 5.00 |
| 273 bp |  |  | 2.08 |  |  |  |  | 1.67 |  |  |  |  |  | 2.08 |  |  |
| 274 bp |  |  |  |  |  |  |  |  |  |  |  | 47.92 |  | 4.17 |  |  |
| 275 bp |  |  | 2.08 |  |  |  | 4.84 | 15.00 | 2.94 |  |  | 18.75 |  |  | 1.67 |  |
| 276 bp | 4.17 | 4.17 | 2.08 | 8.00 | 4.00 | 10.00 | 24.19 | 6.67 | 27.94 | 10.00 | 1.85 | 14.58 |  | 39.58 |  |  |
| 277 bp |  |  |  |  | 2.00 | 34.29 | 64.52 | 41.67 | 29.41 |  |  | 6.25 |  | 4.17 | 63.33 | 33.33 |
| 278 bp | 52.08 | 79.17 | 87.50 | 54.00 | 58.00 | 2.86 |  |  | 4.41 | 55.71 | 62.96 |  | 64.58 | 8.33 |  |  |
| 279 bp |  |  |  |  |  | 10.00 |  | 6.67 | 4.41 | 5.71 | 5.56 |  |  | 2.08 | 10.00 |  |
| 280 bp | 8.33 | 4.17 | 2.08 | 20.00 |  |  |  | 3.33 | 1.47 |  |  |  |  |  |  |  |
| 281 bp |  |  | 2.08 |  |  | 2.86 |  | 6.67 |  |  |  |  |  |  | 3.33 |  |
| 282 bp |  |  |  |  | 4.00 |  |  |  |  |  |  |  | 2.08 |  |  |  |
| 287 bp |  |  |  |  |  |  |  |  | 1.47 |  |  |  |  |  |  |  |
| 293 bp |  |  |  |  |  |  |  |  | 1.47 |  |  |  |  |  |  |  |
| OarFCB20 | BJ | DZ | HW | NB | UW | CDM | CN | JY | MG | YL | ZT | EB | WG | YW | CM | QH |
| 77 bp |  |  |  | 2.00 |  |  |  |  |  |  |  |  |  |  |  |  |
| 83 bp |  |  |  | 2.00 |  |  |  |  |  |  |  |  |  |  |  |  |
| 85 bp | 2.08 |  |  |  | 8.00 | 1.43 |  |  |  |  |  |  |  | 2.08 | 8.33 |  |
| 88 bp |  |  |  |  | 2.00 |  |  |  |  |  |  |  |  |  |  |  |
| 94 bp | 10.42 | 14.58 | 10.87 | 8.00 | 24.00 |  | 4.00 |  | 10.71 | 4.69 | 5.36 | 27.08 | 10.42 | 8.33 | 10.42 | 13.79 |
| 96 bp |  | 6.25 | 2.17 | 20.00 | 4.00 | 11.43 | 2.00 |  |  | 12.50 | 35.71 |  |  | 18.75 | 22.92 | 6.90 |
| 97 bp | 31.25 |  |  |  |  |  | 4.00 | 26.32 | 28.57 |  |  | 4.17 | 14.58 | 8.33 |  |  |
| 98 bp |  |  |  |  |  | 64.29 |  |  |  |  |  |  |  |  | 22.92 | 13.79 |
| 99 bp | 27.08 | 54.17 | 56.52 | 54.00 | 58.00 |  | 62.00 | 60.53 | 50.00 | 25.00 | 35.71 | 25.00 | 56.25 | 43.75 |  |  |
| 101 bp | 20.83 | 10.42 | 26.09 | 8.00 | 2.00 | 7.14 | 10.00 | 5.26 |  | 3.13 | 5.36 | 43.75 | 6.25 | 10.42 | 33.33 | 65.52 |
| 103 bp |  |  |  |  |  |  |  | 5.26 |  |  | 5.36 |  |  | 2.08 | 2.08 |  |
| 105 bp | 4.17 | 14.58 | 4.35 | 6.00 |  | 1.43 | 16.00 |  | 10.71 | 1.56 | 5.36 |  | 8.33 | 4.17 |  |  |
| 106 bp |  |  |  |  | 2.00 |  | 2.00 |  |  |  |  |  |  |  |  |  |
| 107 bp | 2.08 |  |  |  |  |  |  | 2.63 |  |  |  |  |  |  |  |  |
| 122 bp | 2.08 |  |  |  |  |  |  |  |  |  |  |  | 2.08 |  |  |  |
| 123 bp |  |  |  |  |  |  |  |  |  | 1.56 | 1.79 |  |  |  |  |  |
| 124 bp |  |  |  |  |  | 10.00 |  |  |  | 32.81 | 1.79 |  | 2.08 |  |  |  |
| 126 bp |  |  |  |  |  | 2.86 |  |  |  | 18.75 | 1.79 |  |  |  |  |  |
| 129 bp |  |  |  |  |  |  |  |  |  |  | 1.79 |  |  |  |  |  |
| 137 bp |  |  |  |  |  | 1.43 |  |  |  |  |  |  |  |  |  |  |
| 141 bp |  |  |  |  |  |  |  |  |  |  |  |  |  | 2.08 |  |  |
| SRCRSP7 | BJ | DZ | HW | NB | UW | CDM | CN | JY | MG | YL | ZT | EB | WG | YW | CM | QH |
| 116 bp |  |  |  |  |  |  |  |  |  |  |  |  |  |  | 3.45 |  |
| 117 bp |  |  |  |  |  |  | 1.61 |  |  |  |  |  |  |  |  |  |
| 118 bp |  |  |  |  |  |  |  |  | 1.47 |  |  |  |  |  | 6.90 | 3.33 |
| 119 bp |  |  |  |  |  |  |  | 1.67 |  |  |  |  |  |  |  |  |
| 120 bp |  | 6.25 | 27.08 | 14.00 | 23.91 |  |  |  |  | 1.52 | 5.56 | 22.92 |  | 18.75 |  |  |
| 121 bp | 10.42 |  |  |  |  |  | 8.06 | 8.33 | 2.94 |  |  | 4.17 | 35.42 |  |  |  |
| 122 bp |  |  |  |  |  |  | 1.61 |  | 5.88 |  |  |  |  |  |  |  |
| 123 bp |  |  | 4.17 |  |  |  |  | 5.00 | 4.41 |  | 1.85 |  |  |  | 3.45 |  |
| 124 bp | 83.33 | 64.58 | 41.67 | 76.00 | 69.57 | 92.86 | 16.13 | 30.00 | 27.94 | 62.12 | 72.22 | 68.75 | 60.42 | 75.00 | 75.86 | 51.67 |
| 125 bp |  |  |  |  |  |  | 58.06 | 41.67 | 33.82 |  |  |  |  |  |  |  |
| 126 bp |  | 29.17 | 16.67 | 6.00 | 6.52 | 7.14 |  | 3.33 | 5.88 | 22.73 | 1.85 |  |  | 6.25 | 10.34 | 45.00 |
| 127 bp | 6.25 |  | 2.08 |  |  |  | 14.52 | 8.33 | 16.18 | 13.64 | 18.52 |  | 4.17 |  |  |  |
| 128 bp |  |  |  | 4.00 |  |  |  |  |  |  |  |  |  |  |  |  |
| 129 bp |  |  | 6.25 |  |  |  |  |  | 1.47 |  |  |  |  |  |  |  |
| 130 bp |  |  | 2.08 |  |  |  |  | 1.67 |  |  |  |  |  |  |  |  |
| 135 bp |  |  |  |  |  |  |  |  |  |  |  | 4.17 |  |  |  |  |
| ILSTS029 | BJ | DZ | HW | NB | UW | CDM | CN | JY | MG | YL | ZT | EB | WG | YW | CM | QH |
| 146 bp |  |  | 2.17 |  |  |  |  |  |  |  |  |  |  |  |  |  |
| 147 bp |  |  | 2.17 |  |  |  |  |  |  |  |  |  |  | 4.35 |  |  |
| 148 bp |  |  | 2.17 |  |  |  |  |  |  |  |  | 2.08 |  | 2.17 |  |  |
| 150 bp |  |  | 2.17 |  | 2.00 |  | 3.45 |  |  |  |  |  |  |  | 6.25 |  |
| 151 bp | 91.67 | 72.92 | 39.13 | 64.00 | 88.00 | 73.61 | 13.79 | 5.00 | 42.86 | 92.42 | 67.86 | 91.67 | 95.83 | 52.17 | 62.50 | 92.86 |
| 152 bp |  |  | 2.17 |  |  |  | 74.14 | 70.00 | 57.14 |  |  |  |  | 2.17 |  |  |
| 153 bp |  | 2.08 | 6.52 |  |  |  |  |  |  |  |  |  |  |  | 3.13 |  |
| 154 bp |  |  | 15.22 |  |  |  |  |  |  |  |  |  |  | 2.17 |  |  |
| 155 bp |  |  |  |  |  |  |  |  |  |  |  |  |  | 2.17 |  |  |
| 158 bp |  |  | 2.17 |  |  |  |  |  |  |  |  |  |  |  |  |  |
| 159 bp |  |  | 2.17 | 2.00 |  |  |  | 5.00 |  |  | 5.36 |  |  | 8.70 |  |  |
| 161 bp |  | 8.33 |  | 4.00 | 10.00 | 2.78 |  |  |  | 1.52 | 25.00 | 2.08 | 2.08 | 2.17 | 3.13 |  |
| 162 bp |  |  | 2.17 |  |  |  |  |  |  |  |  |  |  |  |  |  |
| 163 bp | 4.17 |  | 2.17 | 6.00 |  |  | 3.45 | 12.50 |  |  |  |  |  |  |  |  |
| 164 bp |  |  |  |  |  |  |  | 2.50 |  |  |  |  |  |  |  |  |
| 165 bp |  |  |  | 2.00 |  | 4.17 | 5.17 | 5.00 |  |  |  | 4.17 | 2.08 | 10.87 |  |  |
| 171 bp |  |  | 2.17 |  |  |  |  |  |  |  |  |  |  |  | 12.50 |  |
| 173 bp |  |  |  |  |  |  |  |  |  |  |  |  |  |  | 3.13 |  |
| 174 bp |  |  |  |  |  |  |  |  |  |  |  |  |  |  | 3.13 |  |
| 176 bp |  | 16.67 | 15.22 | 16.00 |  | 15.28 |  |  |  | 6.06 | 1.79 |  |  | 6.52 |  | 7.14 |
| 177 bp | 2.08 |  |  |  |  |  |  |  |  |  |  |  |  | 4.35 |  |  |
| 178 bp |  |  | 2.17 | 4.00 |  | 4.17 |  |  |  |  |  |  |  |  | 6.25 |  |
| 179 bp |  |  |  |  |  |  |  |  |  |  |  |  |  | 2.17 |  |  |
| 180 bp | 2.08 |  |  | 2.00 |  |  |  |  |  |  |  |  |  |  |  |  |
| SPS113 | BJ | DZ | HW | NB | UW | CDM | CN | JY | MG | YL | ZT | EB | WG | YW | CM | QH |
| 129 bp |  |  |  |  |  |  |  |  |  |  |  |  |  |  | 2.00 |  |
| 131 bp |  |  |  |  |  |  |  |  |  |  |  |  |  |  | 10.00 |  |
| 132 bp |  |  |  |  |  |  |  |  |  | 1.47 |  |  |  |  |  |  |
| 135 bp |  |  |  |  |  |  |  |  | 1.47 | 2.94 |  |  |  |  | 2.00 |  |
| 136 bp |  | 4.17 |  |  | 16.00 |  |  |  |  |  |  |  |  |  |  |  |
| 137 bp | 41.67 | 37.50 | 31.25 | 34.00 | 24.00 | 45.83 | 56.45 | 51.67 | 54.41 | 17.65 | 39.29 | 35.42 | 45.83 | 29.17 | 24.00 | 20.69 |
| 139 bp | 2.08 | 31.25 | 22.92 | 24.00 | 6.00 | 16.67 | 12.90 | 13.33 | 23.53 | 5.88 | 17.86 |  |  | 4.17 | 32.00 | 41.38 |
| 140 bp |  |  |  |  | 8.00 |  |  |  | 1.47 |  |  |  |  |  |  |  |
| 141 bp | 33.33 | 22.92 | 39.58 | 36.00 | 34.00 | 8.33 | 25.81 | 30.00 | 13.24 |  | 7.14 | 10.42 | 45.83 | 41.67 | 10.00 | 5.17 |
| 142 bp |  |  |  |  |  |  |  |  |  |  |  | 4.17 |  |  |  |  |
| 143 bp | 8.33 | 4.17 |  | 4.00 | 8.00 | 15.28 | 3.23 |  | 2.94 | 22.06 | 17.86 | 2.08 | 6.25 | 4.17 | 2.00 | 6.90 |
| 144 bp |  |  | 2.08 |  |  | 1.39 |  |  |  | 5.88 |  | 10.42 |  | 2.08 |  |  |
| 145 bp |  |  |  |  |  | 2.78 |  |  | 1.47 |  | 1.79 |  |  |  | 16.00 | 8.62 |
| 146 bp | 6.25 |  | 4.17 |  | 2.00 | 8.33 | 1.61 |  |  | 23.53 | 5.36 | 2.08 | 2.08 | 6.25 |  |  |
| 147 bp |  |  |  |  |  |  |  |  |  |  | 3.57 |  |  |  | 2.00 |  |
| 148 bp | 8.33 |  |  |  | 2.00 |  |  |  | 1.47 | 11.76 | 3.57 | 35.42 |  | 12.50 |  |  |
| 149 bp |  |  |  |  |  |  |  |  |  | 2.94 |  |  |  |  |  |  |
| 150 bp |  |  |  |  |  | 1.39 |  |  |  | 4.41 |  |  |  |  |  |  |
| 151 bp |  |  |  |  |  |  |  |  |  | 1.47 |  |  |  |  |  |  |
| 154 bp |  |  |  | 2.00 |  |  |  | 5.00 |  |  | 3.57 |  |  |  |  |  |
| 156 bp |  |  |  |  |  |  |  |  |  |  |  |  |  |  |  | 17.24 |
| CSRD247 | BJ | DZ | HW | NB | UW | CDM | CN | JY | MG | YL | ZT | EB | WG | YW | CM | QH |
| 217 bp |  |  |  |  |  |  |  |  |  |  |  |  | 22.92 |  |  |  |
| 220 bp |  | 8.33 | 2.08 | 2.00 | 10.00 |  | 6.45 | 5.56 | 5.88 |  |  | 2.08 |  | 14.58 | 10.00 | 43.10 |
| 221 bp | 16.67 |  | 2.08 |  |  | 8.57 |  |  |  | 4.55 | 11.11 |  | 10.42 |  | 2.00 |  |
| 224 bp |  |  |  |  |  |  |  |  | 1.47 |  |  |  |  |  |  |  |
| 226 bp |  |  |  |  |  |  |  | 3.70 |  |  |  |  |  |  |  |  |
| 227 bp | 27.08 |  |  |  |  |  |  |  |  |  |  |  | 6.25 |  |  |  |
| 228 bp |  |  |  |  |  |  |  |  | 1.47 |  |  |  |  |  |  |  |
| 229 bp | 6.25 |  |  |  |  |  |  |  |  |  |  |  | 10.42 |  |  |  |
| 231 bp | 16.67 |  |  |  |  |  |  | 3.70 | 4.41 |  |  |  | 43.75 |  |  |  |
| 232 bp |  |  |  |  | 4.00 |  |  |  | 1.47 |  |  | 4.17 |  |  |  |  |
| 233 bp | 33.33 |  | 2.08 |  |  |  | 9.68 | 7.41 | 4.41 |  |  | 22.92 | 6.25 | 29.17 | 6.00 |  |
| 234 bp |  | 4.17 | 2.08 | 6.00 | 10.00 |  |  |  | 1.47 |  |  | 2.08 |  | 2.08 |  |  |
| 235 bp |  | 10.42 | 2.08 | 6.00 | 4.00 | 12.86 | 27.42 | 18.52 | 51.47 | 18.18 | 3.70 | 37.50 |  | 22.92 | 18.00 | 3.45 |
| 236 bp |  | 12.50 | 4.17 | 14.00 | 32.00 |  |  |  | 2.94 |  |  |  |  |  |  |  |
| 237 bp |  | 18.75 | 58.33 | 10.00 | 14.00 | 61.43 | 1.61 |  | 2.94 | 42.42 | 57.41 | 2.08 |  |  | 14.00 | 10.34 |
| 238 bp |  |  |  |  | 2.00 |  | 1.61 |  | 2.94 |  |  |  |  |  |  |  |
| 239 bp |  |  |  |  |  |  | 6.45 | 29.63 | 1.47 |  |  |  |  |  | 10.00 | 3.45 |
| 240 bp |  |  |  |  |  |  |  | 1.85 | 2.94 |  |  | 2.08 |  |  |  |  |
| 241 bp |  | 4.17 | 2.08 | 4.00 |  |  | 14.52 | 14.81 |  | 3.03 |  | 12.50 |  | 18.75 | 4.00 |  |
| 242 bp |  | 4.17 |  |  | 6.00 |  |  |  |  |  |  | 4.17 |  | 6.25 |  |  |
| 243 bp |  | 14.58 | 2.08 | 4.00 |  | 7.14 | 30.65 | 14.81 | 13.24 | 1.52 | 5.56 | 10.42 |  | 6.25 | 2.00 |  |
| 244 bp |  | 6.25 |  | 14.00 | 8.00 | 8.57 | 1.61 |  |  | 10.61 |  |  |  |  | 26.00 | 39.66 |
| 245 bp |  | 16.67 | 20.83 | 40.00 | 10.00 |  |  |  | 1.47 | 16.67 | 20.37 |  |  |  |  |  |
| 246 bp |  |  |  |  |  | 1.43 |  |  |  |  | 1.85 |  |  |  | 8.00 |  |
| 247 bp |  |  | 2.08 |  |  |  |  |  |  |  |  |  |  |  |  |  |
| 250 bp |  |  |  |  |  |  |  |  |  | 3.03 |  |  |  |  |  |  |
| SRCRSP5 | BJ | DZ | HW | NB | UW | CDM | CN | JY | MG | YL | ZT | EB | WG | YW | CM | QH |
| 153 bp |  |  |  |  |  |  |  |  |  |  |  |  |  |  | 3.45 |  |
| 154 bp |  |  |  |  |  |  |  |  |  |  |  | 2.17 |  |  |  |  |
| 159 bp |  |  |  |  |  |  |  |  |  |  |  |  |  |  | 1.72 |  |
| 161 bp |  |  |  |  |  |  |  |  |  |  |  | 2.17 |  |  | 1.72 |  |
| 162 bp |  |  |  |  |  |  |  |  |  |  |  |  |  | 4.17 |  |  |
| 163 bp | 6.25 |  | 4.17 |  |  |  |  |  |  |  | 3.85 |  |  |  | 12.07 | 21.15 |
| 164 bp |  |  |  |  |  |  | 5.36 |  | 5.88 |  |  |  |  |  |  |  |
| 165 bp | 12.50 |  | 10.42 | 4.17 | 18.75 | 8.33 | 14.29 | 5.00 | 11.76 | 5.00 | 1.92 | 28.26 | 10.42 | 16.67 | 12.07 |  |
| 166 bp |  |  |  |  |  |  | 5.36 | 2.50 | 5.88 |  |  | 2.17 |  |  |  |  |
| 167 bp | 18.75 | 43.75 | 25.00 | 18.75 | 43.75 | 13.89 | 41.07 | 37.50 | 41.18 | 5.00 | 19.23 | 10.87 | 47.92 | 16.67 | 22.41 | 34.62 |
| 168 bp |  | 20.83 |  |  |  |  |  | 2.50 |  |  |  | 6.52 |  |  |  | 1.92 |
| 169 bp |  | 14.58 | 2.08 | 35.42 | 14.58 | 1.39 | 17.86 | 35.00 | 20.59 | 45.00 |  | 8.70 |  | 18.75 | 20.69 | 26.92 |
| 170 bp | 25.00 | 10.42 | 2.08 | 2.08 |  | 13.89 |  | 2.50 |  | 45.00 | 63.46 | 19.57 | 14.58 | 4.17 |  |  |
| 171 bp |  |  | 2.08 | 6.25 | 8.33 |  | 3.57 | 5.00 | 5.88 |  | 1.92 | 2.17 |  | 4.17 | 20.69 | 7.69 |
| 172 bp | 12.50 |  | 4.17 | 4.17 |  | 6.94 |  | 5.00 | 2.94 |  |  | 4.35 | 8.33 | 4.17 |  |  |
| 173 bp |  |  |  | 2.08 |  |  |  |  |  |  |  | 2.17 |  |  | 1.72 | 1.92 |
| 174 bp |  |  | 2.08 |  |  |  |  |  |  |  |  |  |  |  |  |  |
| 175 bp | 4.17 | 6.25 | 8.33 |  |  |  | 7.14 |  |  |  |  |  | 4.17 | 2.08 |  | 5.77 |
| 176 bp |  |  |  |  |  | 5.56 | 1.79 | 5.00 |  |  |  |  |  |  |  |  |
| 177 bp |  |  | 4.17 | 4.17 | 4.17 |  | 3.57 |  | 2.94 |  |  |  |  | 8.33 | 1.72 |  |
| 178 bp | 14.58 | 2.08 | 33.33 | 2.08 | 10.42 | 47.22 |  |  | 2.94 |  | 5.77 | 10.87 | 14.58 | 18.75 |  |  |
| 179 bp |  |  |  | 2.08 |  |  |  |  |  |  |  |  |  |  | 1.72 |  |
| 180 bp | 6.25 |  |  | 4.17 |  |  |  |  |  |  | 3.85 |  |  | 2.08 |  |  |
| 182 bp |  | 2.08 | 2.08 | 10.42 |  |  |  |  |  |  |  |  |  |  |  |  |
| 184 bp |  |  |  | 4.17 |  | 2.78 |  |  |  |  |  |  |  |  |  |  |
| MAF209 | BJ | DZ | HW | NB | UW | CDM | CN | JY | MG | YL | ZT | EB | WG | YW | CM | QH |
| 98 bp |  |  |  |  |  |  |  |  |  |  |  |  |  |  |  | 1.92 |
| 99 bp |  |  |  |  |  |  | 16.13 |  | 2.94 |  |  | 4.17 |  |  |  |  |
| 100 bp | 35.42 | 43.75 | 15.22 | 16.00 | 30.00 | 19.44 | 16.13 |  | 14.71 | 30.00 | 48.08 | 43.75 | 50.00 | 10.42 | 34.48 | 48.08 |
| 101 bp |  |  |  |  |  |  | 54.84 |  | 32.35 |  |  | 2.08 |  | 2.08 | 13.79 | 7.69 |
| 102 bp | 60.42 | 56.25 | 84.78 | 84.00 | 70.00 | 80.56 | 12.90 | 100.00 | 50.00 | 70.00 | 51.92 | 50.00 | 50.00 | 79.17 | 51.72 | 42.31 |
| 104 bp | 4.17 |  |  |  |  |  |  |  |  |  |  |  |  | 8.33 |  |  |
| SRCRSP8 | BJ | DZ | HW | NB | UW | CDM | CN | JY | MG | YL | ZT | EB | WG | YW | CM | QH |
| 218 bp |  | 12.50 |  |  |  |  | 6.45 |  | 1.47 |  |  |  |  |  |  |  |
| 219 bp |  |  |  |  |  | 2.78 | 1.61 |  |  | 1.43 | 3.57 | 50.00 |  | 4.17 | 5.56 |  |
| 220 bp | 14.58 |  |  |  |  |  |  |  |  |  |  |  |  |  |  |  |
| 226 bp |  | 2.08 |  | 2.00 | 2.00 |  |  |  |  |  |  |  |  |  |  |  |
| 227 bp |  | 6.25 |  |  | 4.00 |  | 8.06 | 1.79 | 1.47 |  | 1.79 | 12.50 |  | 2.08 | 1.85 |  |
| 228 bp | 4.17 | 22.92 | 79.17 | 18.00 | 16.00 | 4.17 | 32.26 | 30.36 | 30.88 | 1.43 |  |  | 8.33 | 8.33 |  |  |
| 229 bp |  |  |  |  | 2.00 |  |  |  | 1.47 | 2.86 | 8.93 | 2.08 |  | 27.08 | 5.56 | 3.70 |
| 230 bp | 33.33 | 8.33 |  | 20.00 |  | 9.72 | 9.68 | 7.14 | 8.82 | 21.43 | 8.93 |  | 35.42 | 4.17 | 27.78 | 46.30 |
| 231 bp |  | 2.08 |  |  |  | 1.39 |  |  |  |  | 1.79 | 8.33 |  | 16.67 |  |  |
| 232 bp | 8.33 |  |  | 4.00 |  | 13.89 |  |  | 14.71 | 18.57 | 10.71 |  | 4.17 |  | 25.93 | 18.52 |
| 233 bp |  |  |  |  |  |  | 1.61 |  | 5.88 | 4.29 | 12.50 | 14.58 |  |  |  |  |
| 234 bp |  |  |  | 4.00 |  | 18.06 |  |  | 8.82 | 28.57 | 21.43 |  |  |  | 1.85 |  |
| 235 bp |  |  |  |  |  |  |  |  | 2.94 |  | 1.79 |  |  |  |  |  |
| 236 bp |  | 31.25 | 18.75 | 36.00 | 72.00 |  | 33.87 | 39.29 | 23.53 |  | 1.79 |  |  | 4.17 |  |  |
| 237 bp |  | 2.08 |  |  | 2.00 | 5.56 | 1.61 |  |  | 7.14 | 3.57 | 8.33 |  | 20.83 |  |  |
| 238 bp | 33.33 | 2.08 |  | 4.00 |  | 38.89 |  | 1.79 |  | 11.43 | 17.86 |  | 50.00 |  | 20.37 | 22.22 |
| 239 bp |  |  |  |  |  |  |  |  |  |  |  |  |  | 6.25 |  |  |
| 240 bp |  |  |  |  |  |  |  |  |  | 2.86 | 3.57 |  |  |  |  |  |
| 241 bp | 4.17 |  |  |  |  |  |  |  |  |  |  | 4.17 |  |  |  |  |
| 242 bp |  | 6.25 |  |  |  |  |  |  |  |  | 1.79 |  |  |  | 1.85 |  |
| 243 bp |  | 2.08 |  |  |  |  |  |  |  |  |  |  |  | 2.08 |  |  |
| 244 bp |  |  |  |  |  |  |  |  |  |  |  |  |  |  | 3.70 |  |
| 246 bp |  | 2.08 | 2.08 | 12.00 |  |  | 3.23 | 16.07 |  |  |  |  |  |  |  |  |
| 247 bp |  |  |  |  |  |  | 1.61 | 3.57 |  |  |  |  |  | 2.08 |  |  |
| 249 bp | 2.08 |  |  |  | 2.00 | 5.56 |  |  |  |  |  |  |  |  | 5.56 | 9.26 |
| 250 bp |  |  |  |  |  |  |  |  |  |  |  |  |  | 2.08 |  |  |
| 251 bp |  |  |  |  |  |  |  |  |  |  |  |  | 2.08 |  |  |  |
| SRCRSP9 | BJ | DZ | HW | NB | UW | CDM | CN | JY | MG | YL | ZT | EB | WG | YW | CM | QH |
| 109 bp |  |  |  |  |  |  | 1.61 |  | 5.88 |  |  | 35.42 |  | 10.42 |  |  |
| 110 bp |  |  |  |  |  |  |  | 3.33 |  |  |  |  |  |  |  |  |
| 113 bp |  |  |  |  |  |  |  | 15.00 | 2.94 |  |  |  |  |  |  |  |
| 114 bp |  |  |  |  |  |  |  | 8.33 |  |  |  |  |  |  |  |  |
| 115 bp |  |  |  |  |  |  |  | 3.33 |  |  |  |  |  |  |  |  |
| 116 bp |  | 2.08 |  |  |  |  | 17.74 | 30.00 | 17.65 |  |  | 4.17 |  |  | 5.00 |  |
| 117 bp |  |  |  |  |  |  | 3.23 | 5.00 | 1.47 |  |  | 50.00 |  | 47.92 |  |  |
| 118 bp |  |  |  | 41.67 | 45.83 | 5.56 |  | 1.67 |  |  | 1.92 |  |  |  | 3.33 |  |
| 119 bp |  |  |  |  |  |  | 1.61 | 1.67 | 1.47 |  |  | 8.33 |  | 25.00 |  |  |
| 120 bp |  |  |  |  |  | 1.39 | 3.23 |  | 7.35 | 16.18 | 13.46 |  |  | 6.25 | 8.33 |  |
| 121 bp | 2.08 | 12.50 |  |  |  |  |  |  |  | 7.35 | 1.92 |  |  |  |  |  |
| 122 bp | 2.08 |  |  |  |  |  |  |  |  |  |  |  |  |  |  |  |
| 123 bp |  |  | 16.67 | 47.92 | 50.00 | 6.94 | 48.39 | 28.33 | 45.59 |  | 7.69 |  |  | 2.08 |  |  |
| 124 bp |  |  |  |  |  |  |  |  |  |  |  |  | 25.00 |  | 1.67 |  |
| 125 bp | 35.42 | 56.25 | 60.42 | 10.42 | 4.17 | 52.78 | 22.58 | 3.33 | 10.29 | 50.00 | 46.15 | 2.08 | 39.58 | 8.33 | 20.00 |  |
| 126 bp |  |  |  |  |  |  |  |  | 1.47 |  |  |  |  |  |  |  |
| 127 bp | 8.33 | 10.42 | 14.58 |  |  | 19.44 | 1.61 |  | 4.41 | 2.94 | 3.85 |  | 12.50 |  | 11.67 | 21.67 |
| 128 bp | 2.08 |  |  |  |  |  |  |  |  |  |  |  | 2.08 |  |  |  |
| 129 bp | 8.33 | 2.08 | 2.08 |  |  | 1.39 |  |  |  | 10.29 | 11.54 |  |  |  | 5.00 |  |
| 131 bp | 18.75 | 4.17 |  |  |  | 8.33 |  |  |  | 1.47 |  |  | 8.33 |  | 5.00 | 28.33 |
| 133 bp | 8.33 | 10.42 | 6.25 |  |  | 2.78 |  |  |  | 4.41 | 11.54 |  | 10.42 |  | 40.00 | 50.00 |
| 136 bp | 14.58 | 2.08 |  |  |  | 1.39 |  |  |  | 7.35 | 1.92 |  | 2.08 |  |  |  |
| 138 bp |  |  |  |  |  |  |  |  | 1.47 |  |  |  |  |  |  |  |
| SRCRSP15 | BJ | DZ | HW | NB | UW | CDM | CN | JY | MG | YL | ZT | EB | WG | YW | CM | QH |
| 177 bp |  |  |  |  |  |  | 1.67 | 8.70 | 15.00 |  |  |  |  |  |  |  |
| 179 bp |  |  |  |  |  |  |  | 4.35 |  |  |  |  |  |  |  |  |
| 181 bp |  |  |  |  |  |  |  |  |  |  |  |  |  |  | 4.26 |  |
| 182 bp |  |  |  |  |  |  |  |  |  | 3.03 |  |  |  |  |  |  |
| 184 bp |  |  |  |  |  |  |  | 2.17 |  |  |  |  |  |  |  |  |
| 185 bp | 2.08 | 16.67 | 18.18 | 2.08 | 30.00 | 12.86 | 16.67 | 4.35 | 17.50 | 1.52 | 13.46 | 2.38 | 25.00 |  | 21.28 | 13.46 |
| 186 bp |  |  |  |  |  |  |  |  |  |  |  |  |  |  | 4.26 |  |
| 187 bp | 83.33 | 70.83 | 81.82 | 77.08 | 56.00 | 87.14 | 78.33 | 69.57 | 67.50 | 84.85 | 78.85 | 11.90 | 62.50 | 2.17 | 40.43 | 69.23 |
| 188 bp |  |  |  |  |  |  | 3.33 |  |  |  |  |  |  |  |  |  |
| 189 bp | 12.50 | 12.50 |  | 16.67 | 12.00 |  |  | 10.87 |  | 3.03 | 5.77 |  | 8.33 |  | 27.66 |  |
| 191 bp | 2.08 |  |  |  |  |  |  |  |  | 7.58 | 1.92 |  |  |  |  | 17.31 |
| 193 bp |  |  |  | 2.08 |  |  |  |  |  |  |  |  |  | 2.17 |  |  |
| 195 bp |  |  |  | 2.08 | 2.00 |  |  |  |  |  |  |  |  |  | 2.13 |  |
| 196 bp |  |  |  |  |  |  |  |  |  |  |  |  | 4.17 |  |  |  |
| 204 bp |  |  |  |  |  |  |  |  |  |  |  |  |  | 2.17 |  |  |
| 206 bp |  |  |  |  |  |  |  |  |  |  |  | 7.14 |  | 45.65 |  |  |
| 208 bp |  |  |  |  |  |  |  |  |  |  |  |  |  | 30.43 |  |  |
| 209 bp |  |  |  |  |  |  |  |  |  |  |  | 11.90 |  | 4.35 |  |  |
| 210 bp |  |  |  |  |  |  |  |  |  |  |  | 64.29 |  | 13.04 |  |  |
| 211 bp |  |  |  |  |  |  |  |  |  |  |  | 2.38 |  |  |  |  |
| TCRVB6 | BJ | DZ | HW | NB | UW | CDM | CN | JY | MG | YL | ZT | EB | WG | YW | CM | QH |
| 216 bp |  |  |  |  |  |  | 1.61 |  | 1.61 |  |  |  |  |  |  |  |
| 223 bp |  |  |  |  |  |  |  |  | 1.61 |  |  |  |  |  |  |  |
| 224 bp |  |  |  |  |  |  |  |  | 1.61 |  |  |  |  |  |  |  |
| 226 bp |  |  |  |  |  |  |  |  | 1.61 |  |  |  |  |  |  |  |
| 228 bp |  |  |  |  |  |  | 50.00 | 16.00 | 12.90 |  | 1.79 |  |  | 4.17 |  |  |
| 229 bp |  | 2.08 | 2.17 | 4.00 | 2.00 | 2.78 |  |  | 3.23 |  |  | 4.17 |  | 12.50 |  | 7.41 |
| 230 bp |  |  |  |  | 22.00 |  | 1.61 | 4.00 |  |  |  | 2.08 |  | 10.42 | 1.85 | 1.85 |
| 231 bp | 47.92 | 25.00 | 67.39 | 6.00 | 38.00 | 6.94 | 3.23 |  |  |  |  |  | 58.33 |  | 24.07 | 51.85 |
| 232 bp |  |  |  |  |  |  | 3.23 | 12.00 | 1.61 |  |  |  |  | 6.25 |  |  |
| 233 bp |  | 2.08 | 6.52 |  |  | 13.89 | 1.61 |  |  |  |  |  |  | 2.08 | 3.70 | 9.26 |
| 234 bp |  |  |  |  |  |  |  | 2.00 | 4.84 |  |  |  |  |  |  |  |
| 235 bp | 4.17 | 2.08 | 2.17 | 26.00 |  | 5.56 | 3.23 |  |  | 1.43 | 7.14 | 4.17 | 4.17 | 4.17 | 5.56 | 7.41 |
| 236 bp |  |  |  |  |  |  | 1.61 | 4.00 | 1.61 |  |  | 4.17 |  | 6.25 |  |  |
| 237 bp | 2.08 | 2.08 |  | 8.00 |  | 8.33 |  | 4.00 | 1.61 |  | 1.79 | 4.17 | 2.08 |  | 3.70 |  |
| 238 bp |  |  |  |  |  |  | 1.61 |  |  |  |  | 4.17 |  | 18.75 | 3.70 | 3.70 |
| 239 bp | 12.50 |  |  | 8.00 | 4.00 | 1.39 |  |  |  |  | 1.79 |  |  |  | 16.67 | 14.81 |
| 240 bp |  | 25.00 | 2.17 |  |  |  |  |  | 1.61 |  |  |  |  | 2.08 |  |  |
| 241 bp |  |  |  |  |  | 2.78 |  |  |  |  |  |  |  |  | 3.70 |  |
| 243 bp |  |  |  |  |  |  |  |  | 16.13 |  |  |  |  |  | 1.85 |  |
| 244 bp | 2.08 |  |  |  |  |  |  |  |  | 7.14 |  |  |  |  |  |  |
| 245 bp |  |  |  |  |  |  |  | 16.00 | 1.61 |  |  |  |  |  |  |  |
| 246 bp | 6.25 | 2.08 |  | 4.00 | 2.00 | 4.17 |  |  | 3.23 | 48.57 | 16.07 |  | 6.25 |  | 3.70 |  |
| 247 bp |  |  |  |  |  |  |  |  | 1.61 |  |  |  |  |  |  |  |
| 248 bp |  |  |  | 4.00 |  | 1.39 |  | 4.00 |  |  |  |  |  |  |  |  |
| 249 bp |  |  |  |  |  |  | 30.65 | 38.00 | 32.26 |  |  | 10.42 |  | 2.08 |  |  |
| 250 bp |  |  |  |  |  | 8.33 |  |  |  | 11.43 | 32.14 | 66.67 |  | 31.25 | 3.70 |  |
| 251 bp |  |  |  |  | 6.00 |  | 1.61 |  | 6.45 |  |  |  |  |  |  |  |
| 252 bp | 25.00 | 39.58 | 19.57 | 40.00 | 26.00 | 44.44 |  |  | 4.84 | 30.00 | 39.29 |  | 29.17 |  | 25.93 | 3.70 |
| 254 bp |  |  |  |  |  |  |  |  |  | 1.43 |  |  |  |  | 1.85 |  |
| MAF70 | BJ | DZ | HW | NB | UW | CDM | CN | JY | MG | YL | ZT | EB | WG | YW | CM | QH |
| 70 bp |  |  |  |  |  |  |  |  |  |  |  |  |  | 2.17 |  |  |
| 75 bp |  |  |  |  |  |  |  |  |  |  |  |  |  | 2.17 |  |  |
| 90 bp |  |  |  |  |  |  |  |  |  |  |  |  |  | 2.17 |  |  |
| 96 bp |  |  |  |  |  |  |  |  |  |  |  |  |  | 10.87 |  |  |
| 97 bp |  |  |  |  |  |  |  |  |  |  |  | 95.83 |  | 82.61 |  |  |
| 134 bp |  |  |  |  |  |  |  |  |  |  |  |  | 4.17 |  |  |  |
| 136 bp |  |  |  |  |  |  |  |  |  |  | 7.14 |  |  |  | 7.69 | 9.26 |
| 137 bp | 2.17 |  | 14.58 | 4.00 | 2.00 |  |  |  |  |  |  | 4.17 |  |  |  |  |
| 138 bp |  |  |  |  |  |  |  |  |  |  |  |  |  |  | 5.77 |  |
| 140 bp |  |  |  |  |  |  |  |  | 1.47 |  |  |  |  |  |  |  |
| 141 bp |  |  |  |  |  | 1.47 |  |  |  |  | 5.36 |  | 2.08 |  | 9.62 |  |
| 143 bp | 15.22 |  |  |  | 14.00 | 4.41 | 1.67 | 1.67 | 2.94 |  |  |  | 10.42 |  | 7.69 |  |
| 145 bp |  | 2.08 | 4.17 | 2.00 | 2.00 | 13.24 |  | 20.00 | 13.24 |  |  |  | 4.17 |  |  |  |
| 147 bp | 36.96 | 50.00 | 58.33 | 36.00 | 28.00 | 25.00 | 51.67 | 48.33 | 51.47 | 7.58 | 35.71 |  | 27.08 |  | 13.46 | 18.52 |
| 149 bp | 4.35 | 4.17 |  | 2.00 | 2.00 | 1.47 |  |  |  |  |  |  |  |  | 3.85 | 55.56 |
| 151 bp | 19.57 | 20.83 | 12.50 | 8.00 | 30.00 | 14.71 | 26.67 | 6.67 | 16.18 | 3.03 | 10.71 |  | 22.92 |  | 7.69 | 1.85 |
| 152 bp |  |  |  |  |  |  | 3.33 | 1.67 |  |  |  |  |  |  |  |  |
| 153 bp | 21.74 | 22.92 | 10.42 | 48.00 | 22.00 | 33.82 | 15.00 | 21.67 | 13.24 | 89.39 | 37.50 |  | 27.08 |  | 28.85 | 14.81 |
| 154 bp |  |  |  |  |  |  | 1.67 |  |  |  |  |  |  |  |  |  |
| 155 bp |  |  |  |  |  | 5.88 |  |  | 1.47 |  | 1.79 |  | 2.08 |  | 15.38 |  |
| 157 bp |  |  |  |  |  |  |  |  |  |  | 1.79 |  |  |  |  |  |
| OarFCB48 | BJ | DZ | HW | NB | UW | CDM | CN | JY | MG | YL | ZT | EB | WG | YW | CM | QH |
| 137 bp |  |  |  |  |  |  |  |  |  | 2.86 |  |  |  |  |  |  |
| 139 bp |  |  |  |  |  |  |  |  |  |  |  |  |  |  | 1.92 |  |
| 145 bp |  |  |  |  |  |  |  |  |  |  |  |  |  |  | 1.92 |  |
| 146 bp |  |  |  |  |  |  |  |  |  |  |  |  |  | 2.08 |  |  |
| 147 bp |  |  |  |  |  |  |  | 1.67 | 1.47 |  |  |  |  |  |  |  |
| 148 bp |  |  |  |  |  | 1.47 |  |  | 2.94 |  |  |  |  |  | 7.69 |  |
| 149 bp |  |  |  |  |  |  | 1.61 |  | 7.35 |  |  |  |  |  |  |  |
| 150 bp | 2.08 |  |  | 10.00 | 4.00 | 7.35 | 3.23 | 5.00 | 13.24 | 2.86 | 7.14 |  | 4.17 | 2.08 | 19.23 |  |
| 151 bp |  |  |  |  |  |  |  |  |  |  |  |  |  |  |  | 3.57 |
| 152 bp | 39.58 | 33.33 | 47.92 | 44.00 | 40.00 | 38.24 | 46.77 | 25.00 | 47.06 | 61.43 | 62.50 | 27.08 | 43.75 | 52.08 | 7.69 | 44.64 |
| 153 bp |  |  |  |  | 2.00 |  |  |  | 2.94 |  |  |  |  |  |  |  |
| 154 bp | 2.08 | 18.75 | 22.92 | 4.00 | 20.00 | 7.35 | 11.29 | 5.00 | 7.35 | 2.86 | 3.57 |  | 14.58 | 2.08 | 17.31 |  |
| 155 bp |  |  |  |  |  |  |  |  | 2.94 |  |  |  |  |  |  |  |
| 156 bp |  |  |  | 2.00 |  |  | 1.61 | 3.33 | 2.94 |  | 3.57 |  | 4.17 | 2.08 | 3.85 | 7.14 |
| 157 bp |  |  |  |  |  |  |  | 1.67 | 2.94 |  |  |  |  |  |  |  |
| 158 bp | 8.33 |  | 8.33 | 6.00 |  | 1.47 |  | 6.67 |  | 1.43 | 5.36 |  |  | 2.08 | 7.69 | 28.57 |
| 159 bp |  |  |  |  |  | 5.88 |  |  | 1.47 | 11.43 |  |  |  |  |  |  |
| 160 bp | 45.83 | 45.83 | 20.83 | 22.00 | 34.00 | 32.35 | 20.97 | 15.00 | 5.88 | 5.71 | 12.50 | 72.92 | 33.33 | 27.08 | 13.46 | 16.07 |
| 161 bp |  |  |  |  |  | 2.94 |  | 5.00 |  |  | 3.57 |  |  |  | 7.69 |  |
| 162 bp | 2.08 |  |  | 12.00 |  |  | 8.06 | 25.00 |  |  |  |  |  | 4.17 |  |  |
| 163 bp |  |  |  |  |  |  |  | 1.67 |  |  |  |  |  |  | 1.92 |  |
| 165 bp |  |  |  |  |  |  |  | 1.67 |  | 1.43 |  |  |  |  | 5.77 |  |
| 166 bp |  | 2.08 |  |  |  |  | 1.61 | 1.67 |  |  |  |  |  |  |  |  |
| 167 bp |  |  |  |  |  | 2.94 |  |  |  | 10.00 | 1.79 |  |  | 4.17 | 3.85 |  |
| 168 bp |  |  |  |  |  |  | 4.84 | 1.67 | 1.47 |  |  |  |  | 2.08 |  |  |
| OarAE54 | BJ | DZ | HW | NB | UW | CDM | CN | JY | MG | YL | ZT | EB | WG | YW | CM | QH |
| 119 bp |  |  |  | 4.00 |  |  |  |  |  | 1.52 |  |  |  |  | 1.92 |  |
| 120 bp |  | 2.08 | 2.17 |  |  | 1.52 | 3.45 |  |  | 1.52 |  |  |  |  | 15.38 |  |
| 121 bp | 2.08 | 4.17 | 2.17 | 40.00 | 32.00 | 4.55 |  |  | 6.90 | 43.94 | 7.14 | 4.17 |  | 6.25 | 42.31 | 42.86 |
| 122 bp | 25.00 | 43.75 | 39.13 |  |  | 22.73 | 32.76 | 31.82 | 43.10 | 18.18 | 71.43 | 35.42 | 39.58 | 14.58 |  |  |
| 124 bp |  |  | 2.17 |  |  |  |  |  |  |  |  |  |  |  |  |  |
| 125 bp |  |  |  |  |  |  |  | 4.55 | 6.90 |  |  | 2.08 |  |  | 3.85 |  |
| 126 bp | 41.67 | 12.50 | 28.26 | 16.00 | 48.00 | 27.27 | 31.03 | 18.18 | 13.79 | 7.58 |  | 27.08 | 39.58 | 35.42 | 13.46 | 21.43 |
| 127 bp |  |  |  |  |  |  |  |  | 3.45 |  |  |  |  |  | 1.92 |  |
| 128 bp | 2.08 |  |  | 4.00 |  | 4.55 | 3.45 |  |  | 3.03 |  |  |  |  | 1.92 |  |
| 130 bp | 2.08 | 10.42 |  | 8.00 |  | 3.03 | 1.72 | 15.91 | 1.72 | 1.52 |  |  |  |  |  | 7.14 |
| 131 bp |  |  |  |  |  |  |  | 4.55 |  |  |  |  |  |  |  |  |
| 132 bp |  |  |  |  | 8.00 |  |  |  |  | 1.52 | 1.79 |  |  | 2.08 | 9.62 |  |
| 133 bp | 8.33 | 2.08 | 4.35 |  |  |  | 1.72 |  |  |  |  | 2.08 | 6.25 | 6.25 |  |  |
| 135 bp | 4.17 |  |  |  | 4.00 |  | 5.17 |  |  |  |  | 2.08 | 2.08 | 4.17 | 1.92 | 3.57 |
| 136 bp |  |  |  |  |  |  | 3.45 |  | 3.45 |  |  | 2.08 |  | 6.25 |  |  |
| 137 bp | 12.50 | 14.58 | 6.52 | 12.00 | 8.00 | 27.27 | 8.62 | 25.00 | 17.24 | 4.55 | 10.71 | 20.83 | 12.50 | 22.92 | 5.77 | 21.43 |
| 138 bp |  |  |  |  |  |  |  |  |  |  |  |  |  | 2.08 |  |  |
| 139 bp |  | 2.08 |  | 12.00 |  | 7.58 | 1.72 |  | 3.45 | 12.12 | 8.93 | 4.17 |  |  |  |  |
| 141 bp | 2.08 | 8.33 | 13.04 | 4.00 |  | 1.52 | 6.90 |  |  | 4.55 |  |  |  |  |  |  |
| 143 bp |  |  | 2.17 |  |  |  |  |  |  |  |  |  |  |  |  |  |
| 144 bp |  |  |  |  |  |  |  |  |  |  |  |  |  |  | 1.92 | 3.57 |
| TGLA53 | BJ | DZ | HW | NB | UW | CDM | CN | JY | MG | YL | ZT | EB | WG | YW | CM | QH |
| 123 bp |  |  |  |  |  |  |  |  | 2.94 |  |  |  |  |  |  |  |
| 130 bp |  |  |  |  |  |  |  |  |  |  |  |  |  |  | 3.45 |  |
| 133 bp |  |  |  |  |  |  |  | 1.72 | 1.47 |  |  |  |  |  |  |  |
| 134 bp |  |  |  |  |  |  |  |  |  |  |  |  |  |  | 3.45 | 1.67 |
| 135 bp | 2.08 |  |  | 2.00 |  |  |  |  | 1.47 | 4.55 | 3.57 |  |  |  | 1.72 |  |
| 136 bp |  |  |  |  |  |  |  |  |  |  |  |  |  |  | 3.45 |  |
| 137 bp | 4.17 | 12.50 | 10.42 | 12.00 | 2.17 | 6.06 | 27.42 | 24.14 | 17.65 | 13.64 | 7.14 | 43.75 | 2.08 | 15.22 | 5.17 | 8.33 |
| 138 bp |  |  |  |  |  |  |  |  | 7.35 |  |  |  |  |  |  |  |
| 139 bp | 31.25 |  |  | 22.00 | 19.57 | 15.15 | 24.19 | 10.34 | 25.00 | 1.52 | 5.36 |  | 20.83 | 6.52 | 15.52 | 11.67 |
| 140 bp |  | 18.75 | 27.08 |  |  |  | 1.61 | 1.72 | 2.94 |  |  |  |  |  |  | 1.67 |
| 141 bp |  |  |  |  |  |  |  |  | 1.47 |  | 1.79 |  |  |  |  |  |
| 142 bp | 2.08 | 2.08 | 4.17 | 8.00 | 4.35 |  |  | 5.17 | 1.47 |  | 7.14 |  | 4.17 |  | 8.62 |  |
| 143 bp |  |  |  |  |  |  | 3.23 |  | 1.47 |  |  |  |  |  |  |  |
| 144 bp | 4.17 |  |  | 2.00 | 2.17 |  |  |  | 1.47 | 3.03 |  | 6.25 | 4.17 | 4.35 | 13.79 | 18.33 |
| 145 bp |  |  |  |  |  |  |  |  | 1.47 |  |  |  |  |  |  |  |
| 146 bp | 56.25 |  |  | 46.00 | 58.70 | 78.79 | 30.65 | 41.38 | 25.00 | 59.09 | 67.86 | 47.92 | 58.33 | 67.39 | 36.21 | 45.00 |
| 147 bp |  | 52.08 | 50.00 |  |  |  | 3.23 | 5.17 | 1.47 |  |  |  |  |  |  |  |
| 148 bp |  |  |  |  |  |  |  |  |  | 18.18 | 5.36 |  |  | 4.35 |  |  |
| 149 bp |  | 12.50 | 8.33 | 4.00 | 6.52 |  | 9.68 | 5.17 | 2.94 |  |  | 2.08 | 6.25 | 2.17 | 1.72 |  |
| 150 bp |  |  |  |  |  |  |  |  |  |  |  |  |  |  |  | 1.67 |
| 151 bp |  |  |  | 2.00 | 4.35 |  |  |  | 1.47 |  |  |  | 4.17 |  | 6.90 | 1.67 |
| 153 bp |  |  |  |  |  |  |  | 1.72 |  |  |  |  |  |  |  | 10.00 |
| 156 bp |  |  |  |  |  |  |  | 1.72 |  |  |  |  |  |  |  |  |
| 158 bp |  |  |  |  | 2.17 |  |  |  |  |  |  |  |  |  |  |  |
| 159 bp |  |  |  |  |  |  |  |  | 1.47 |  |  |  |  |  |  |  |
| 160 bp |  | 2.08 |  | 2.00 |  |  |  |  |  |  | 1.79 |  |  |  |  |  |
| 161 bp |  |  |  |  |  |  |  | 1.72 | 1.47 |  |  |  |  |  |  |  |
