# Supplementary material for: Genetic diversity of the Chinese goat in the littoral zone of the Yangtze River as assessed by microsatellite and mtDNA
Source: Ecol Evol. 2018 Apr 24;8(10):5111–23. doi: 10.1002/ece3.4100 (PMC5980450; doi:10.1002/ece3.4100)
Supplement: Supplementary file 2 [file ECE3-8-5111-s002.doc]

Appendix S2. STRUCTURE_harvester Analysis Result


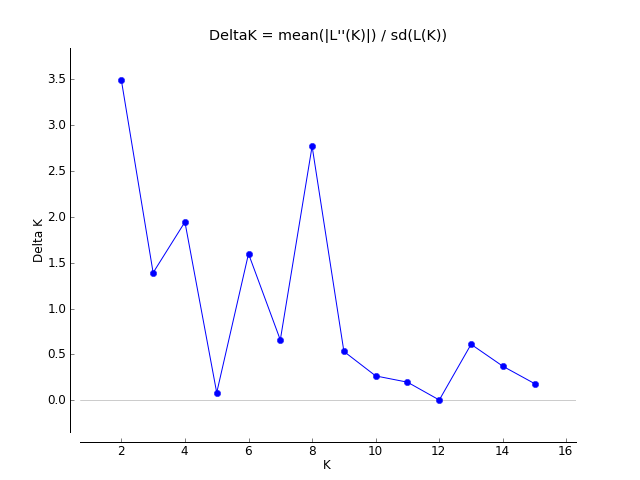


Figure 1. Delta *K* pattern of STRUCTURE-harvester

Table 1 The Evanno table of STRUCTURE-harvester for each *K*

| *K* | Reps | Mean LnP(*K*) | Stdev LnP(*K*) | Ln'(*K*) | |Ln''(*K*)| | Delta *K* |
| --- | --- | --- | --- | --- | --- | --- |
| 1 | 100 | -36357.128000 | 1.544039 | — | — | — |
| **2** | **100** | **-33677.195000** | **322.013690** | **2679.933000** | **1122.384000** | **3.485516** |
| 3 | 100 | -32119.646000 | 200.987313 | 1557.549000 | 279.256000 | 1.389421 |
| 4 | 100 | -30841.353000 | 201.477759 | 1278.293000 | 391.075000 | 1.941033 |
| 5 | 100 | -29954.135000 | 1596.742232 | 887.218000 | 134.860000 | 0.084459 |
| 6 | 100 | -28932.057000 | 256.850351 | 1022.078000 | 409.838000 | 1.595630 |
| 7 | 100 | -28319.817000 | 173.953555 | 612.240000 | 114.570000 | 0.658624 |
| **8** | **100** | **-27822.147000** | **132.863391** | **497.670000** | **367.346000** | **2.764840** |
| 9 | 100 | -27691.823000 | 1848.304911 | 130.324000 | 985.698000 | 0.533298 |
| 10 | 100 | -28547.197000 | 6876.976231 | -855.374000 | 1835.367000 | 0.266886 |
| 11 | 100 | -27567.204000 | 4617.111681 | 979.993000 | 918.075000 | 0.198842 |
| 12 | 100 | -27505.286000 | 2392.232097 | 61.918000 | 12.162000 | 0.005084 |
| 13 | 100 | -27431.206000 | 1917.243257 | 74.080000 | 1171.379000 | 0.610970 |
| 14 | 100 | -28528.505000 | 4339.929586 | -1097.299000 | 1615.585000 | 0.372261 |
| 15 | 100 | -28010.219000 | 3425.611922 | 518.286000 | 626.777000 | 0.182968 |
| 16 | 100 | -28118.710000 | 3413.404181 | -108.491000 | — | — |
