# Supplementary material for: Genetic diversity of the Chinese goat in the littoral zone of the Yangtze River as assessed by microsatellite and mtDNA
Source: Ecol Evol. 2018 Apr 24;8(10):5111–23. doi: 10.1002/ece3.4100 (PMC5980450; doi:10.1002/ece3.4100)
Supplement: Supplementary file 3 [file ECE3-8-5111-s003.doc]

Appendix S3. Polymorphism of mtDNA D-loop haplotypes by 117 SNPs

117 SNPs from 15737 to16188 referenced as AF533441, the Location of each SNPs in reference sequences as Table 1.

Table 1. the Location of each SNPs which constructed the Haplotype in this study

| SNP number | Location in Reference sequences (bp) | References |
| --- | --- | --- |
| 1 | 15742 | AF533441 |
| 2 | 15743 | AF533441 |
| 3 | 15744 | AF533441 |
| 4 | 15747 | AF533441 |
| 5 | 15756 | AF533441 |
| 6 | 15759 | AF533441 |
| 7 | 15763 | AF533441 |
| 8 | 15771 | AF533441 |
| 9 | 15776 | AF533441 |
| 10 | 15777 | AF533441 |
| 11 | 15779 | AF533441 |
| 12 | 15780 | AF533441 |
| 13 | 15783 | AF533441 |
| 14 | 15797 | AF533441 |
| 15 | 15800 | AF533441 |
| 16 | 15803 | AF533441 |
| 17 | 15805 | AF533441 |
| 18 | 15806 | AF533441 |
| 19 | 15807 | AF533441 |
| 20 | 15809 | AF533441 |
| 21 | 15810 | AF533441 |
| 22 | 15811 | AF533441 |
| 23 | 15812 | AF533441 |
| 24 | 15813 | AF533441 |
| 25 | 15822 | AF533441 |
| 26 | 15823 | AF533441 |
| 27 | 15832 | AF533441 |
| 28 | 15833 | AF533441 |
| 29 | 15834 | AF533441 |
| 30 | 15835 | AF533441 |
| 31 | 15836 | AF533441 |
| 32 | 15842 | AF533441 |
| 33 | 15843 | AF533441 |
| 34 | 15844 | AF533441 |
| 35 | 15862 | AF533441 |
| 36 | 15864 | AF533441 |
| 37 | 15866 | AF533441 |
| 38 | 15870 | AF533441 |
| 39 | 15873 | AF533441 |
| 40 | 15885 | AF533441 |
| 41 | 15887 | AF533441 |
| 42 | 15888 | AF533441 |
| 43 | 15889 | AF533441 |
| 44 | 15891 | AF533441 |
| 45 | 15893 | AF533441 |
| 46 | 15896 | AF533441 |
| 47 | 15898 | AF533441 |
| 48 | 15909 | AF533441 |
| 49 | 15910 | AF533441 |
| 50 | 15911 | AF533441 |
| 51 | 15912 | AF533441 |
| 52 | 15913 | AF533441 |
| 53 | 15915 | AF533441 |
| 54 | 15920 | AF533441 |
| 55 | 15921 | AF533441 |
| 56 | 15929 | AF533441 |
| 57 | 15930 | AF533441 |
| 58 | 15945 | AF533441 |
| 59 | 15947 | AF533441 |
| 60 | 15950 | AF533441 |
| 61 | 15965 | AF533441 |
| 62 | 15967 | AF533441 |
| 63 | 15969 | AF533441 |
| 64 | 15970 | AF533441 |
| 65 | 15972 | AF533441 |
| 66 | 15973 | AF533441 |
| 67 | 15974 | AF533441 |
| 68 | 15975 | AF533441 |
| 69 | 15976 | AF533441 |
| 70 | 15977 | AF533441 |
| 71 | 15978 | AF533441 |
| 72 | 15981 | AF533441 |
| 73 | 15982 | AF533441 |
| 74 | 15983 | AF533441 |
| 75 | 15984 | AF533441 |
| 76 | 15992 | AF533441 |
| 77 | 15994 | AF533441 |
| 78 | 15999 | AF533441 |
| 79 | 16000 | AF533441 |
| 80 | 16001 | AF533441 |
| 81 | 16002 | AF533441 |
| 82 | 16003 | AF533441 |
| 83 | 16006 | AF533441 |
| 84 | 16007 | AF533441 |
| 85 | 16009 | AF533441 |
| 86 | 16010 | AF533441 |
| 87 | 16011 | AF533441 |
| 88 | 16015 | AF533441 |
| 89 | 16019 | AF533441 |
| 90 | 16022 | AF533441 |
| 91 | 16026 | AF533441 |
| 92 | 16027 | AF533441 |
| 93 | 16028 | AF533441 |
| 94 | 16037 | AF533441 |
| 95 | 16038 | AF533441 |
| 96 | 16040 | AF533441 |
| 97 | 16042 | AF533441 |
| 98 | 16043 | AF533441 |
| 99 | 16045 | AF533441 |
| 100 | 16048 | AF533441 |
| 101 | 16054 | AF533441 |
| 102 | 16060 | AF533441 |
| 103 | 16062 | AF533441 |
| 104 | 16065 | AF533441 |
| 105 | 16066 | AF533441 |
| 106 | 16069 | AF533441 |
| 107 | 16073 | AF533441 |
| 108 | 16081 | AF533441 |
| 109 | 16083 | AF533441 |
| 110 | 16084 | AF533441 |
| 111 | 16112 | AF533441 |
| 112 | 16114 | AF533441 |
| 113 | 16119 | AF533441 |
| 114 | 16147 | AF533441 |
| 115 | 16150 | AF533441 |
| 116 | 16151 | AF533441 |
| 117 | 16174 | AF533441 |


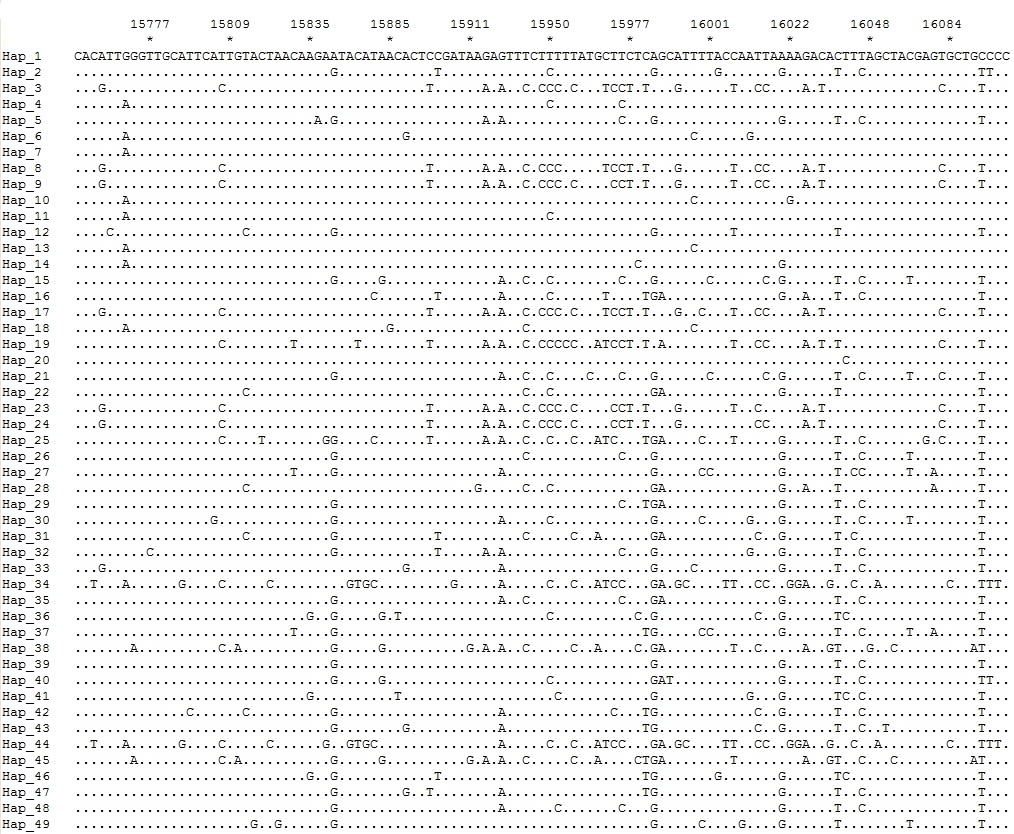

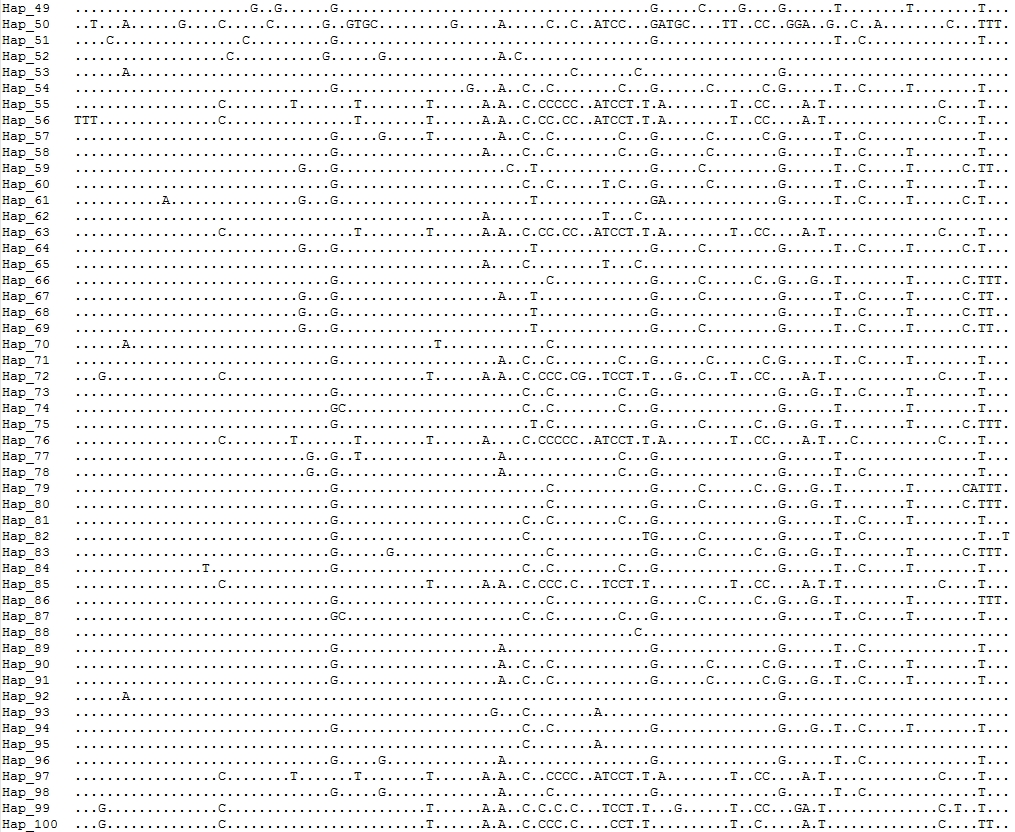


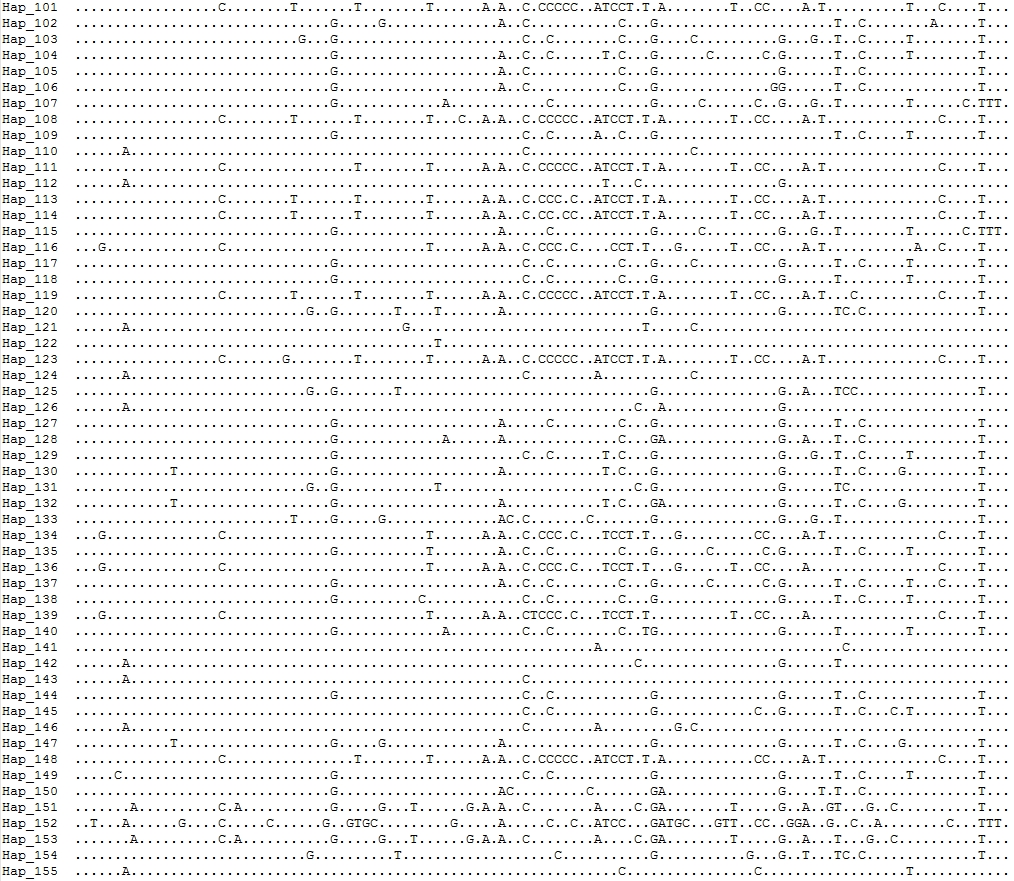


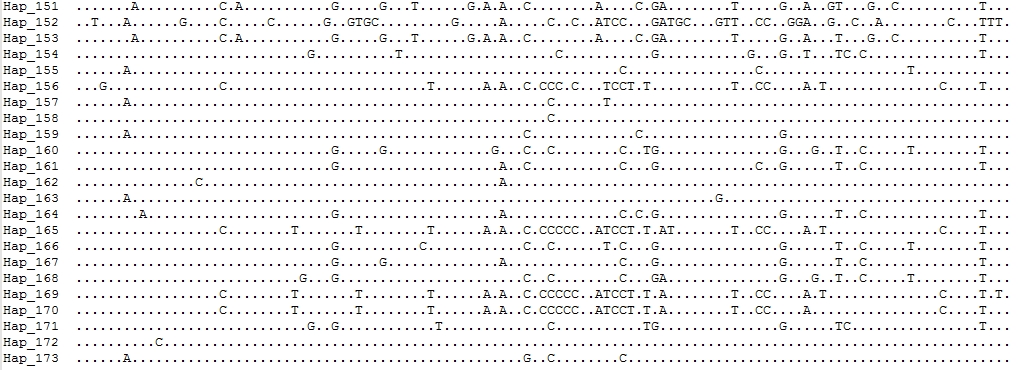


Figure 1. Polymorphism sites [component](http://www.baidu.com/link?url=kQFYgK1RBq4Ilc5DdD95og0aNRL_bxBNjcWDL05NtSgfSYYwm28OlofSCVguVOLPOMPqlCheL_uo3Fufls3TaE79McsmaWIdFvKYk0tsG4u) of 173 haplotypes in this study
