# Supplementary material for: Genetic diversity of the Chinese goat in the littoral zone of the Yangtze River as assessed by microsatellite and mtDNA
Source: Ecol Evol. 2018 Apr 24;8(10):5111–23. doi: 10.1002/ece3.4100 (PMC5980450; doi:10.1002/ece3.4100)
Supplement: Supplementary file 4 [file ECE3-8-5111-s004.doc]

Appendix S4. Frequency of haplotype of 22 goat population using mtDNA variants

| Hap_No. | Nu_Cp | Seq (GCN) |
| --- | --- | --- |
| Hap_1 | 21 | KX660779, KX660792, KX660783, KX660784, MTH1_DQ121577, DQ121583, KU891468, KU891471, KU891475, KU891463, KU891464, KX660974, KX660986, KX660989, KX660994, KX660995, KX660997, KX661000, KX661002, KX660976, KX660979 |
| Hap_2 | 2 | KX660787, KX660790 |
| Hap_3 | 60 | KX660788, KX660789, KX660780, KX660796, KX660800, KX660801 BJ4_KX660782, KX660813, KX660817, KX660819, KX660821, KX660808, KX660824, KX660825, KX660826, DQ089215, DQ089209, KU891488, KU891492, KU891493, KU891480, KU891482, KU891485, KU891486, KU891487, KU891434, KU891435, KU891436, KU891437, KU891438, KU891429, KU891430, KU891431, KU891432, DQ089163, KU891454, KU891455, KU891456, KU891443, KU891447, KX660926, DQ089269, DQ089273, DQ089276, AY860933, AY860924, KU891459, KU891469, KU891474, KX660992, KX661003, KX660980, KX660982, DQ089257, DQ089249, DQ089251, HQ199198, HQ199199, HQ199200, HQ199201 |
| Hap_4 | 7 | KX660791, DQ089222, AY860882, AY860883, HQ199163, HQ199164, HQ199167 |
| Hap_5 | 1 | KX660793 |
| Hap_6 | 3 | KX660794, KX660806, AY860880 |
| Hap_7 | 15 | KX660795, KX660798, AY860884, DQ089211, DQ089242, DQ089243, DQ121580, AY860921, AY860927, KU891470, HQ199109, HQ199162, HQ199160, HQ199165, HQ199168 |
| Hap_8 | 1 | KX660797 |
| Hap_9 | 7 | KX660799,DQ089214, DQ089218, KX660870, KX660898, KX660907, KX660943 |
| Hap_10 | 6 | KX660802, KX660781, DQ089212, DQ089250, DQ089253, DQ089254 |
| Hap_11 | 19 | KX660803, KX660805, KX660786, KX660823, KX660809, DQ089213, DQ089216 CDW14_DQ089217, DQ089219, DQ089220, DQ089221, DQ089210, KU891433, KU891403, AY860931, AY860925, DQ121596, KU891477, HQ199166 |
| Hap_12 | 1 | KX660804 |
| Hap_13 | 4 | KX660785, KU891460, KX660988, KX660996 |
| Hap_14 | 10 | KX660814, KX660828, KU891483, KU891439, KU891452, KU891442, KU891413, KU891417, AY860929, AY860928 |
| Hap_15 | 1 | KX660815 |
| Hap_16 | 1 | KX660816 |
| Hap_17 | 14 | KX660807, KX660811, KU891489, KU891491, KU891481, KU891484, KU891426, KU891441, KU891450, KU891451, KU891457, KU891444, KU891446, HQ199202 |
| Hap_18 | 3 | KX660818, DQ121588, KX660977 |
| Hap_19 | 1 | KX660820 |
| Hap_20 | 4 | KX660822, KU891440, KU891427, AY860926 |
| Hap_21 | 1 | KX660827 |
| Hap_22 | 1 | KX660810 |
| Hap_23 | 1 | KX660812 |
| Hap_24 | 1 | AY860881 |
| Hap_25 | 1 | KX660829 |
| Hap_26 | 1 | KX660838 |
| Hap_27 | 1 | KX660839 |
| Hap_28 | 2 | KX660840, KX660835 |
| Hap_29 | 1 | KX660841 |
| Hap_30 | 1 | KX660842 |
| Hap_31 | 3 | KX660843, KX660844, KX660853 |
| Hap_32 | 1 | KX660845 |
| Hap_33 | 1 | KX660846 |
| Hap_34 | 1 | KX660847 |
| Hap_35 | 1 | KX660830 |
| Hap_36 | 1 | KX660848 |
| Hap_37 | 1 | KX660849 |
| Hap_38 | 1 | KX660850 |
| Hap_39 | 2 | KX660851, DQ121602 |
| Hap_40 | 1 | KX660852 |
| Hap_41 | 2 | KX660854, KX660948 |
| Hap_42 | 1 | KX660855 |
| Hap_43 | 1 | KX660856 |
| Hap_44 | 1 | KX660831 |
| Hap_45 | 1 | KX660857 |
| Hap_46 | 1 | KX660858 |
| Hap_47 | 1 | KX660832 |
| Hap_48 | 1 | KX660833 |
| Hap_49 | 1 | KX660834 |
| Hap_50 | 1 | KX660836 |
| Hap_51 | 1 | KX660837 |
| Hap_52 | 1 | KU891479 |
| Hap_53 | 2 | KU891490, KU891494 |
| Hap_54 | 4 | KX660859, KX660915, KX660917, KX660918 |
| Hap_55 | 26 | KX660868, KX660875, KX660882, KX660884, KX660885, KX660863, KX660867, DQ089176, DQ121530, KX660927, KX660928, KX660936, KX660937, KX660922, DQ089278, DQ089279, DQ089270, DQ089272, KU891406, KU891408, KU891420, KU891423, AY860922, DQ121591, DQ121603, DQ121598 |
| Hap_56 | 1 | KX660869 |
| Hap_57 | 1 | KX660871 |
| Hap_58 | 1 | KX660872 |
| Hap_59 | 1 | KX660873 |
| Hap_60 | 2 | KX660874, KX660864 |
| Hap_61 | 1 | KX660876 |
| Hap_62 | 1 | KX660877 |
| Hap_63 | 5 | KX660860, KX660878, KX660886, KX660887, KX660861 |
| Hap_64 | 1 | KX660879 |
| Hap_65 | 2 | KX660880, KX660883 |
| Hap_66 | 3 | KX660881, KU891414, KU891399 |
| Hap_67 | 1 | KX660862 |
| Hap_68 | 1 | KX660865 |
| Hap_69 | 12 | KX660866, KU891402, DQ089381, DQ089390, DQ089392, DQ089394, DQ089382, DQ089383, DQ089384, DQ089386, DQ089387, DQ089388 |
| Hap_70 | 1 | KU891424 |
| Hap_71 | 10 | KU891425, KU891448, KX660914, KX660931, KX660932, KX660934, KX660939, KX660940, KX660941, KU891418 |
| Hap_72 | 1 | KU891428 |
| Hap_73 | 5 | HQ199110, DQ089167, KX660916, KX660919, KU891415 |
| Hap_74 | 2 | HQ199169, KX660902 |
| Hap_75 | 1 | HQ199170 |
| Hap_76 | 1 | DQ089160 |
| Hap_77 | 1 | DQ089169 |
| Hap_78 | 1 | DQ089170 |
| Hap_79 | 1 | DQ089171 |
| Hap_80 | 2 | DQ089172, KU891398 |
| Hap_81 | 11 | DQ089173, DQ089175, KX660897, KX660901 HG17_KX660904, KX660906, KX660911, KX660912, KX660913, KX660929, KU891410 |
| Hap_82 | 1 | DQ089174 |
| Hap_83 | 2 | DQ089177, DQ089166 |
| Hap_84 | 2 | DQ089161, DQ089165 |
| Hap_85 | 1 | DQ089162 |
| Hap_86 | 1 | DQ089164 |
| Hap_87 | 1 | DQ089168 |
| Hap_88 | 2 | DQ089237, DQ089244 |
| Hap_89 | 3 | DQ121521, DQ121525, DQ121595 |
| Hap_90 | 2 | DQ121531, DQ121534 |
| Hap_91 | 1 | DQ121532 |
| Hap_92 | 1 | DQ121533 |
| Hap_93 | 1 | DQ089238 |
| Hap_94 | 2 | DQ121522, DQ121527 |
| Hap_95 | 1 | DQ089239 |
| Hap_96 | 2 | DQ121523, DQ121524 |
| Hap_97 | 2 | DQ089240, DQ089241 |
| Hap_98 | 1 | DQ121526 |
| Hap_99 | 1 | DQ121528 |
| Hap_100 | 1 | DQ089245 |
| Hap_101 | 2 | DQ121529, KX660933 |
| Hap_102 | 2 | KX660888, KX660892 |
| Hap_103 | 2 | KX660899, KX660900 |
| Hap_104 | 4 | KX660903, KX660890, KX660895, KX660896 |
| Hap_105 | 2 | KX660905, KX660889 |
| Hap_106 | 2 | KX660908, KX660909 |
| Hap_107 | 1 | KX660910 |
| Hap_108 | 2 | KX660891, KX660893 |
| Hap_109 | 1 | KX660894 |
| Hap_110 | 10 | KU891453, KU891445, DQ121586, DQ089280, DQ089271, DQ089274, DQ089275, DQ089277, KU891472, DQ089247 |
| Hap_111 | 7 | KU891458, DQ121581, KX660985, KX660987, KX660981, DQ089256, DQ089258 |
| Hap_112 | 1 | KU891449 |
| Hap_113 | 1 | KX660923 |
| Hap_114 | 2 | KX660924, KX660925 |
| Hap_115 | 1 | KX660930 |
| Hap_116 | 1 | KX660935 |
| Hap_117 | 1 | KX660938 |
| Hap_118 | 1 | KX660942 |
| Hap_119 | 2 | KX660920, KX660921 |
| Hap_120 | 1 | DQ121587 |
| Hap_121 | 1 | DQ121589 |
| Hap_122 | 1 | DQ121590 |
| Hap_123 | 7 | DQ121578, DQ121579, DQ121585, KU891462, KU891465, KX660975, KX661001 |
| Hap_124 | 1 | DQ121582 |
| Hap_125 | 6 | DQ121584, KU891478, KU891461, KU891466, KX660983, KX660990 |
| Hap_126 | 1 | KU891395 |
| Hap_127 | 1 | KU891404 |
| Hap_128 | 1 | KU891405 |
| Hap_129 | 1 | KU891407 |
| Hap_130 | 1 | KU891409 |
| Hap_131 | 2 | KU891411, DQ089395 |
| Hap_132 | 1 | KU891412 |
| Hap_133 | 1 | KU891396 |
| Hap_134 | 1 | KU891416 |
| Hap_135 | 1 | KU891419 |
| Hap_136 | 1 | KU891421 |
| Hap_137 | 2 | KU891422, AY860923 |
| Hap_138 | 5 | KU891397, KU891401, DQ089398, DQ089399, DQ089402 |
| Hap_139 | 1 | KU891400 |
| Hap_140 | 1 | AY860920 |
| Hap_141 | 1 | AY860930 |
| Hap_142 | 1 | AY860932 |
| Hap_143 | 1 | DQ121600 |
| Hap_144 | 1 | DQ121601 |
| Hap_145 | 1 | DQ121592 |
| Hap_146 | 1 | DQ121593 |
| Hap_147 | 1 | DQ121594 |
| Hap_148 | 1 | DQ121597 |
| Hap_149 | 1 | DQ121599 |
| Hap_150 | 13 | KX660944, KX660957, KX660960, KX660961, KX660945, KX660963, KX660965, KX660969, KX660970, KX660971, KX660972, KX660950, KX660951 |
| Hap_151 | 6 | KX660953, KX660956, KX660958, KX660959, KX660946, KX660947 |
| Hap_152 | 8 | KX660954, KX660955, KX660962, KX660964, KX660966, KX660968 , KX660973, KX660952 |
| Hap_153 | 1 | KX660967 |
| Hap_154 | 1 | KX660949 |
| Hap_155 | 1 | KU891473 |
| Hap_156 | 2 | KU891476, KX660999 |
| Hap_157 | 1 | KU891467 |
| Hap_158 | 2 | KX660984, KX660991 |
| Hap_159 | 1 | KX660993 |
| Hap_160 | 2 | KX660998, KX660978 |
| Hap_161 | 1 | DQ089246 |
| Hap_162 | 2 | DQ089255, DQ089248 |
| Hap_163 | 1 | DQ089252 |
| Hap_164 | 3 | DQ089391, DQ089393 , DQ089389 |
| Hap_165 | 1 | DQ089396 |
| Hap_166 | 1 | DQ089397 |
| Hap_167 | 1 | DQ089400 |
| Hap_168 | 1 | DQ089401 |
| Hap_169 | 1 | DQ089403 |
| Hap_170 | 1 | DQ089404 |
| Hap_171 | 1 | DQ089385 |
| Hap_172 | 1 | HQ199161 |
| Hap_173 | 1 | HQ199159 |
